# VEGFR2 activity on myeloid cells mediates immune suppression in the tumor microenvironment

**DOI:** 10.1172/jci.insight.150735

**Published:** 2021-12-08

**Authors:** Yuqing Zhang, Huocong Huang, Morgan Coleman, Arturas Ziemys, Purva Gopal, Syed M. Kazmi, Rolf A. Brekken

**Affiliations:** 1 Hamon Center for Therapeutic Oncology Research,; 2 Department of Surgery,; 3 Cancer Biology Graduate Program, and; 4 Division of Pediatric Hematology and Oncology, Department of Pediatrics, University of Texas (UT) Southwestern, Dallas, Texas, USA.; 5 Program of Mathematics in Medicine, Houston Methodist Research Institute, Houston, Texas, USA.; 6 Department of Pathology and; 7 Division of Hematology and Oncology, Department of Medicine, UT Southwestern, Dallas, Texas, USA.

**Keywords:** Immunology, Oncology, Cancer immunotherapy, Macrophages

## Abstract

Angiogenesis, a hallmark of cancer, is induced by vascular endothelial growth factor–A (hereafter VEGF). As a result, anti-VEGF therapy is commonly used for cancer treatment. Recent studies have found that VEGF expression is also associated with immune suppression in patients with cancer. This connection has been investigated in preclinical and clinical studies by evaluating the therapeutic effect of combining antiangiogenic reagents with immune therapy. However, the mechanisms of how anti-VEGF strategies enhance immune therapy are not fully understood. We and others have shown selective elevation of VEGFR2 expression on tumor-associated myeloid cells in tumor-bearing animals. Here, we investigated the function of VEGFR2^+^ myeloid cells in regulating tumor immunity and found VEGF induced an immunosuppressive phenotype in VEGFR2^+^ myeloid cells, including directly upregulating the expression of programmed cell death 1 ligand 1. Moreover, we found that VEGF blockade inhibited the immunosuppressive phenotype of VEGFR2^+^ myeloid cells, increased T cell activation, and enhanced the efficacy of immune checkpoint blockade. This study highlights the function of VEGFR2 on myeloid cells and provides mechanistic insight on how VEGF inhibition potentiates immune checkpoint blockade.

## Introduction

A malignant tumor that consists of rapidly dividing and growing cancer cells demands a dedicated blood supply to provide nutrients that support the growth and progression of the tumor. Angiogenesis can be induced by various growth factors secreted by cells in the tumor microenvironment. One such factor is vascular endothelial growth factor–A (hereafter referred to as VEGF). VEGF induces endothelial cell survival, proliferation, and migration; thus, VEGF is a major driver of tumor neovascularization (1, 2). The function of VEGF is mediated by binding to its receptor tyrosine kinases (RTKs) VEGFR1 and VEGFR2, with VEGFR2 (gene: *KDR*, *Flk-1*) being the dominant RTK that triggers VEGF-induced angiogenesis (2). Expression of VEGF, VEGFR1, and VEGFR2 is elevated in most solid tumors (2, 3), and due to the importance of tumor angiogenesis, VEGF is an attractive therapeutic target. Multiple strategies have been developed to inhibit the VEGF pathway. Antiangiogenic agents, including small molecule inhibitors and monoclonal antibodies (mAbs) targeting VEGF (e.g., bevacizumab) or VEGFR2 (e.g., ramucirumab), are US Food and Drug Administration (FDA) approved for the treatment of renal cell carcinoma, colorectal cancer, gastric cancer, non-small-cell lung cancer, and a number of other indications (4). However, antiangiogenic strategies used as a single agent generally provide modest efficacy in patients with cancer because of insufficient response, therapy resistance, and tumor adaptation (5–7).

Cancer immunotherapy is a breakthrough in the field of cancer therapy, with strategies disrupting immune checkpoint pathways being particularly effective in a subset of patients (8). Blocking immune checkpoint molecules can reverse tumor immune suppression, activate tumor cell–specific cytotoxic CD8^+^ T cells, and trigger adaptive antitumor immune responses (8). Antagonistic mAbs specific for programmed cell death protein 1 (PD-1) and its ligand, programmed cell death 1 ligand 1 (PD-L1), have shown efficacy and have been approved by the FDA for use in a variety of cancer types (4). However, immunotherapies, mainly immune checkpoint inhibitors (ICIs), are more effective in tumors with preexisting tumor-infiltrating lymphocytes (TILs) and an inflammatory microenvironment; thus, clinical benefit with ICIs is often limited to a fraction of patients with cancer (9). Multiple strategies are being investigated to reduce the immunosuppressive microenvironment of tumors in an effort to enhance the efficacy of ICIs (8, 10). Angiogenesis and immunosuppression can occur in parallel, facilitating tumor development and progression (11). Indeed, multiple angiogenic factors, especially VEGF, have been shown to have immunosuppressive functions, and high VEGF expression is reported to be associated with the suppression of antitumor immune activity in patients with cancer (3, 4). Given the potential immune-stimulatory effects of antiangiogenic therapies, preclinical and clinical studies have investigated the therapeutic effects of combining antiangiogenic reagents with ICIs (4). Therefore, understanding the mechanism of how antiangiogenic and in particular anti-VEGF therapies affect tumor immunity and ICI activity is urgent.

Although VEGFRs were initially considered to be expressed exclusively on endothelial cells, it is now appreciated that VEGFRs are also expressed on other cell types, including some tumor cells, dendritic cells (DCs), tumor-associated macrophages (TAMs), and T cells (12–14). Thus, VEGF can directly influence immune cell phenotype and function. For example, VEGF stimulation of VEGFR2 can inhibit DC maturation, thus reducing tumor neoantigen presentation (15, 16). In addition, VEGF has been reported to directly suppress effector T cell proliferation and activation through VEGFR2 (17). It has also been demonstrated that VEGF contributes to CD8^+^ T cell exhaustion by upregulating immune checkpoint molecules’ expression including PD-1, cytotoxic T lymphocyte–associated antigen 4 (CTLA-4), and T cell immunoglobulin mucin receptor 3 (18). In contrast, VEGF stimulation of VEGFR2 on regulatory T cells (Tregs) results in proliferation (19). Furthermore, VEGF can affect immune cells and the immune system indirectly through modulation of the tumor endothelium (20).

In earlier studies, we and others have shown selective elevation of VEGFR2 expression on myeloid-derived suppressor cells (MDSCs) and macrophages in tumor-bearing animals (3, 12, 21). However, the function of VEGFR2^+^ myeloid cells and their contribution to tumor immunosuppression is not understood. Previously, our lab developed a fully human mAb (r84) specific for VEGF that disrupts VEGF binding to VEGFR2 but does not disrupt VEGF–VEGFR1 interaction (2, 22). Prior work from our group and others demonstrated that r84 alone or in combination with standard therapy controls the growth of primary tumors in multiple indications with limited toxicity (2, 7, 23). Here, by using mouse chimeric r84 (mcr84) that specifically inhibits the mouse VEGF/VEGFR2 axis and a genetic mouse model with VEGFR2-specific ablation on myeloid cells, we investigate the function of VEGFR2^+^ myeloid cells in modulating tumor immunity and provide a comprehensive investigation of how VEGFR2 blockade facilitates an antitumor immune response. We show that VEGFR2 activity on myeloid cells mediates immunosuppression and that inhibition of VEGFR2 signaling results in reduction of PD-L1 expression on myeloid cells and contributes to reinvigorated T cell activation in the tumor microenvironment, which improves response to ICI.

## Results

### Inhibition of VEGF activation of VEGFR2 by mcr84 delays tumor progression and reduces the vascular immune barrier.

**Figure 1:**
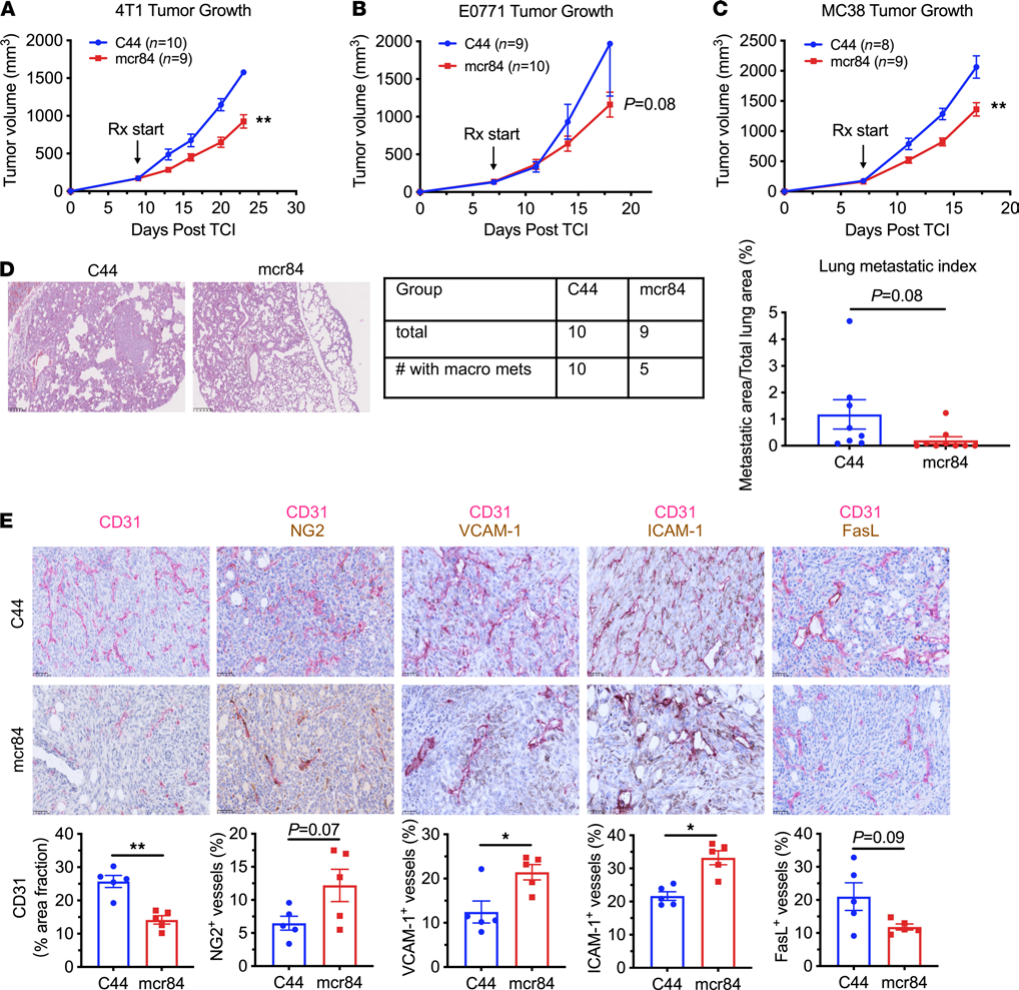
Selective inhibition of VEGF activation of VEGFR2 by mcr84 delays tumor progression and reduces the vascular immune barrier in syngeneic models. (**A**–**C**) In vivo assessment of tumor growth in response to mcr84 treatment in orthotopically or subcutaneously implanted tumors. (**A**) A total of 1 × 10^5^ 4T1 cells (*n* = 9–10/group) were injected orthotopically into 8-week-old BALB/c mice. (**B**) A total of 1 × 10^5^ E0771 cells (*n* = 9–10/group) were injected orthotopically into 8-week-old C57BL/6 mice. (**C**) A total of 1 × 10^5^ MC38 cells were injected subcutaneously into 8-week-old C57BL/6 mice (*n* = 8–9/group). Mice with established tumors (50–150 mm^3^) were treated with control antibody (C44, 250 μg/dose, twice per week) or mcr84 (250 μg/dose, twice per week). Mice were monitored daily and tumor volume was measured twice per week. All mice were sacrificed when tumor volume in the control group reached 2000 mm^3^. Data are displayed as mean ± SEM. **, *P* < 0.01 vs. control, by Welch’s *t* test. (**D**) Lung metastasis burden was evaluated in the 4T1 model. Formalin-fixed, paraffin-embedded (FFPE) lung tissues were sectioned serially at a 150 μm interval. H&E staining was performed to evaluate metastasis. Metastasis index was calculated by metastatic area/total lung area. Representative images of H&E staining are shown. Scale bar: 100 μm (left), 250 μm (right). (**E**) IHC of FFPE 4T1 tumors for CD31, CD31 and neural/glial antigen 2 (NG2), CD31 and vascular cell adhesion protein 1 (VCAM-1), CD31 and intercellular adhesion molecule 1 (ICAM-1), and CD31 and Fas ligand (FasL). Slides were scanned and images were analyzed using NIS Elements (Nikon) and Fiji software. Representative images are shown with CD31 in red and other markers in brown. Scale bar, 50 μm. Quantification is shown. Data are displayed as mean ± SEM (*n* = 5/group). *, *P* < 0.05; **, *P* < 0.005 vs. control, by Welch’s *t* test.

We have previously demonstrated the effect of different anti-VEGF strategies on the growth of breast cancer xenografts and mouse syngeneic models (24). To evaluate the effect of mcr84 on the tumor immune landscape, we studied 4T1 and E0771 breast tumors implanted orthotopically in female C57BL/6 and BALB/c mice, respectively, and MC38 mouse colon tumors implanted subcutaneously in C57BL/6 mice. Mice bearing established tumors were treated with mcr84 or an isotype-matched control IgG (C44) until the tumor volume in the control group reached 2000 mm^3^. In all 3 models, VEGF blockade by mcr84 modestly suppressed tumor growth as a single agent (Figure 1, A–C). mcr84 as a single agent also reduced metastasis to lungs in mice bearing 4T1 tumors (Figure 1D). In addition, we harvested tumors of similar size from these 2 groups for analysis (Supplemental Figure 1, A and B; supplemental material available online with this article; https://doi.org/10.1172/jci.insight.150735DS1).

To determine the effect of mcr84 on angiogenesis, we analyzed tumor microvessel density and vessel maturation by immunohistochemistry (IHC). In 4T1 tumors, overall vascular area, marked by CD31, was decreased significantly by mcr84 compared with control-treated tumors (Figure 1E), consistent with previous studies (24). Because immature blood vessels without pericytes’ support tend to be more vulnerable to anti-VEGF therapies (25), we performed double staining of CD31 and the pericyte marker NG2 and observed that mcr84 increased pericyte coverage of the remaining tumor vessels (Figure 1E), which is typically associated with improved vascular function (26). In addition to nutrient and oxygen delivery, functional blood vessels also facilitate immune cell infiltration, which involves homing of immune cells, adhesion to the endothelium, and diapedesis mediated by chemokines (20). Angiogenic factors can regulate the expression of adhesion molecules or secretion of chemokines by tumor endothelium, which limit T cell attachment to the vessel and efficient T cell infiltration (27). We found that inhibition of VEGF with mcr84 elevated the expression of VCAM-1 and ICAM-1 on tumor endothelium in 4T1 tumors (Figure 1E). We also found that mcr84 reduced microvessel density and consistently upregulated adhesion molecule expression on tumor endothelium in MC38 tumors (Supplemental Figure 1, C and D). These changes in tumor vasculature were confirmed by flow cytometry of 4T1 tumors after short-term treatment (1 week; Supplemental Figure 1E). Furthermore, we and others have reported that VEGF can increase the expression of the death mediator FasL on tumor-associated endothelial cells, and this can reduce T cell infiltration (28, 29). Here, we showed that mcr84 suppressed FasL expression on tumor endothelium in 4T1 tumors (Figure 1E).

Together, these results demonstrate the single-agent effect of mcr84 on control of tumor progression in multiple syngeneic models. Importantly, VEGF blockade effectively inhibited angiogenesis while increasing pericyte coverage and modulating tumor endothelium, resulting in a vasculature that supports immune cell trafficking.

### Expression of VEGFR2 on myeloid cells is elevated specifically in tumor-bearing animals and is associated with an immunosuppressive myeloid phenotype.

Different from VEGFR1, which has been widely acknowledged to be expressed by macrophages and other myeloid cells, myeloid cells express low to no VEGFR2 under normal conditions (12). Previously, we identified a subset of macrophages expressing VEGFR2 only in tumor-bearing animals (12). We found that VEGF was a potent chemoattractant for VEGFR2^+^ macrophages and that selectively inhibiting VEGF binding to VEGFR2 decreased the recruitment of VEGFR2^+^ macrophages (12). Consistent with preclinical studies, CD45^bright^/CD14^+^ monocytes expressing VEGFR2 become dominant in circulating blood from patients with cancer compared with healthy donors (30). Recent studies have extended our findings to multiple indications. In an ovarian tumor model, in contrast to VEGFR1^+^ MDSCs, whose proportion remains unchanged across organs, the frequency of VEGFR2^+^ MDSCs increases significantly in tumors, indicating the importance of VEGFR2 for recruitment of MDSCs (3). However, the functional significance of VEGFR2 expression and activity of myeloid cells remains unclear.

**Figure 2:**
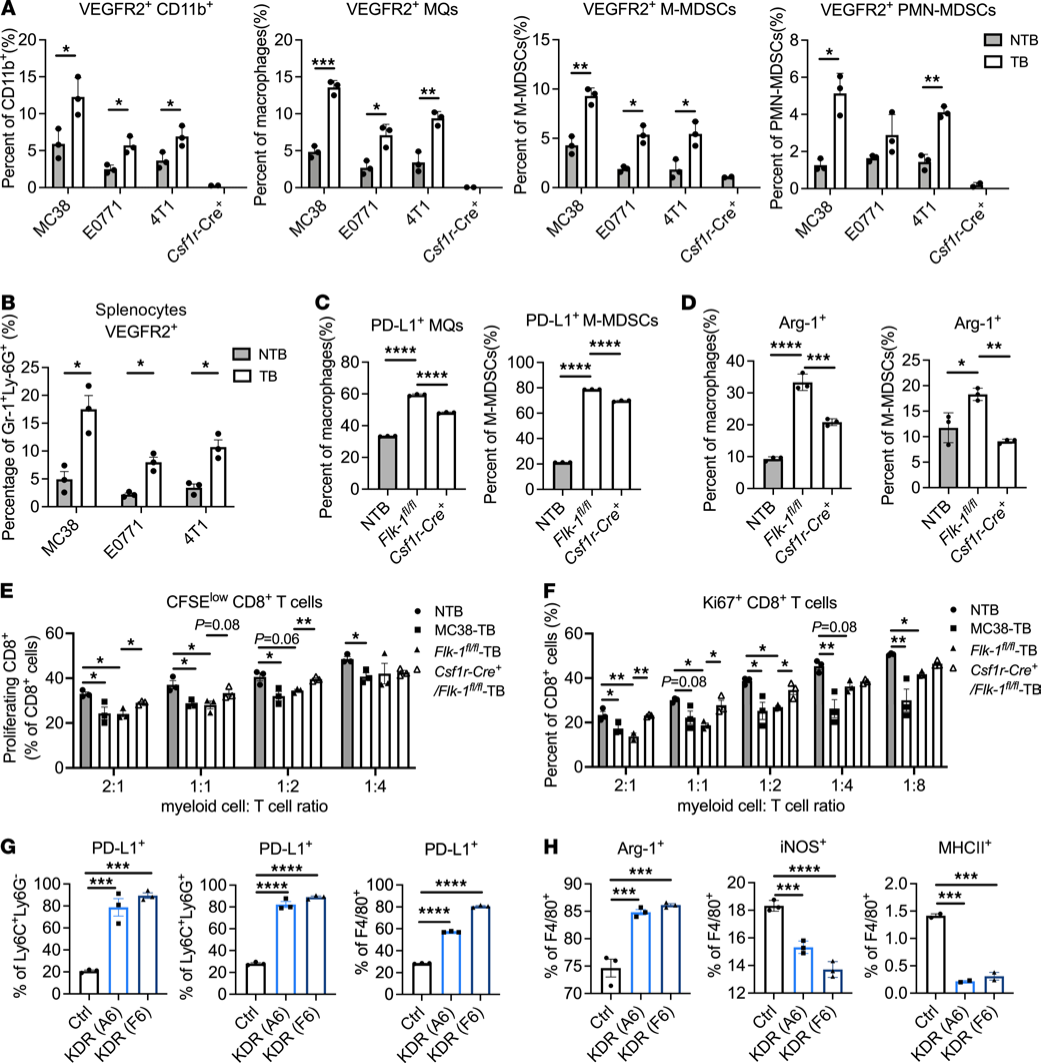
Expression of VEGFR2 on myeloid cells is elevated specifically in tumor-bearing animals and is associated with an immunosuppressive myeloid phenotype. (**A**) BM-derived total myeloid cells, macrophages (MQs), and MDSCs from NTB animals; MC38, E0771, and 4T1 TB animals; and colony-stimulating factor 1 receptor–Cre^+^ *Flk-1^fl/fl^* (*Csf1r*-*Cre*^+^ *Flk-1^fl/fl^*) animals were analyzed for VEGFR2 expression by flow cytometry. (**B**) Gr-1^+^Ly-6G^+^ MDSCs were sorted from splenocytes of NTB mice, MC38, E0771 and 4T1 TB mice and VEGFR2 expression were evaluated by flow cytometry. Data are displayed as mean ± SEM with 3 independent experiments. *, *P* < 0.05; **, *P* < 0.005; ***, *P* < 0.001, by Welch’s *t* test. (**C** and **D**) BM-derived myeloid cells from NTB mice and *Flk-1^fl/fl^* and *Csf1r-Cre^+^ Flk-1^fl/fl^* mice bearing F246-6 breast tumors were analyzed by flow cytometry for PD-L1 and Arg-1 expression. Data are displayed as mean ± SEM with 3 independent experiments. *, *P* < 0.05; **, *P* < 0.005; ***, *P* < 0.001; ****, *P* < 0.0001 by ANOVA with Tukey’s multiple comparisons test (MCT). (**E** and **F**) BM-derived myeloid cells from NTB mice and MC38, *Flk-1^fl/fl^*, and *Csf1r-Cre^+^ Flk-1^fl/fl^* TB mice at day 6 were harvested and added to CD8^+^ T cells at different ratios. Percentages of proliferating CD8^+^ T cells after 72 hours were analyzed by CFSE signal (**E**) or intracellular Ki67 staining (**F**) with flow cytometry. Data are displayed as mean ± SEM with 3 independent experiments. *, *P* < 0.05; **, *P* < 0.005 by ANOVA with Tukey’s MCT. (**G** and **H**) KDR was overexpressed by lentiviral transduction in J774M cells, and clones (A6 and F6) were chosen. J774M-Ctrl and J774M-KDR (A6) as well as J774M-KDR (F6) cells were analyzed for PD-L1 and other myeloid cell markers as indicated by flow cytometry. Data are displayed as mean ± SEM with 3 independent experiments. ***, *P* < 0.001; ****, *P* < 0.0001 by ANOVA with Tukey’s MCT.

We examined the expression level of VEGFR1 and VEGFR2 on bone marrow–derived macrophages (BM-MQs) and bone marrow–derived MDSCs (BM-MDSCs) in syngeneic murine cancer models MC38, E0771, and 4T1 (Supplemental Figure 2A). *Vegfr1* expression was similar in myeloid cells from non-tumor-bearing (NTB) and tumor-bearing (TB) animals; however, *Vegfr2* was elevated on BM-MQs and BM-MDSCs from TB mice (Supplemental Figure 2B). Increased VEGFR2 expression on myeloid cells in TB mice was verified at protein level by flow cytometric analysis of BM-myeloid cells, including total CD11b^+^ myeloid cells, macrophages, monocytic MDSCs (M-MDSCs), and polymorphonuclear MDSCs (PMN-MDSCs) (Figure 2A). BM-myeloid cells from *Csf1r*-*Cre*^+^ *Flk-1^fl/fl^* animals were used as a negative control. In addition, we sorted Gr-1^+^Ly-6G^+^ MDSCs from splenocytes of NTB and TB mice and found VEGFR2 was upregulated on splenic MDSCs from TB animals (Figure 2B). These results are consistent with our prior findings that VEGFR2 is expressed by macrophages in mice bearing pancreatic or breast tumors (12). We further compared the phenotype of BM-myeloid cells from NTB and TB mice. In 4T1 and MC38 models, higher baseline level of PD-L1 was observed in BM-Ly-6C^+^Ly-6G^–^, Ly-6C^+^Ly-6G^+^, and F4/80^+^ myeloid cells (Supplemental Figure 2, C and D). Furthermore, BM-MQs from TB mice showed decreased iNOS expression but an elevated Arginase 1^+^/inducible NOS^+^ (Arg-1^+^/iNOS^+^) ratio (Supplemental Figure 2, E and F). Moreover, after injecting an MMTV-PyMT–derived breast cancer cell line, F246-6, into control *Flk-1^fl/fl^* and *Csf1r*-*Cre*^+^ *Flk-1^fl/fl^* mice, in which *Vegfr2* (*Flk-1*) was specifically ablated in CSF1R^+^ myeloid cells, we harvested the BM from *Cre*^–^ and *Cre*^+^ littermates and differentiated into macrophages or MDSCs in vitro. Interestingly, elevated PD-L1 and Arg-1 expression in BM-MQs and M-MDSCs from TB mice were lowered in those from *Cre*^+^ TB mice (Figure 2, C and D), indicating the involvement of VEGFR2 in mediating PD-L1 and Arg-1 expression.

To demonstrate the immunosuppressive function of VEGFR2^+^ myeloid cells, we cocultured BM-myeloid cells from NTB mice as well as MC38, *Flk-1^fl/fl^*, and *Csf1r*-*Cre*^+^ *Flk-1^fl/fl^* TB mice with CFSE-labeled wild-type CD8^+^ T cells. The proliferation of CD8^+^ T cells was inhibited by BM-myeloid cells from NTB mice in a dose-dependent manner (Supplemental Figure 2G), and the inhibition effect was further enhanced by myeloid cells from TB animals (Figure 2E). However, specific deletion of *Vegfr2* in myeloid cells partially rescued the effect (Figure 2E). Consistent findings were observed by analyzing Ki67 level in proliferating CD8^+^ T cells (Figure 2F).

To further characterize the phenotype of myeloid cells with elevated expression of VEGFR2, we utilized a CD11b^+^Gr-1^+^ MDSC-like cell line, J774M (31), and expressed human VEGFR2, *KDR*, by lentiviral infection. Two clones with high KDR expression were chosen: J774M-KDR (A6) and J774M-KDR (F6; Supplemental Figure 3A). J774M-Ctrl cells were infected with an empty vector. Interestingly, J774M-KDR (A6) and J774M-KDR (F6) cells demonstrated macrophage phenotype with decreased Ly-6C and Ly-6G expression but increased F4/80 expression (Supplemental Figure 3B). Meanwhile, J774M-KDR cells have higher baseline levels of PD-L1 expression on Ly-6C^+^Ly-6G^–^, Ly-6C^+^Ly-6G^+^, and F4/80^+^ subpopulations (Figure 2G). J774M-KDR cells also exhibited an enhanced immunosuppressive phenotype evidenced by elevated Arg-1 expression but decreased iNOS and MHCII expression (Figure 2H). To test the function of the secretome of J774M-KDR, we treated CD8^+^ T cell with conditioned medium harvested from J774M-Ctrl and J774M-KDR cells and found the conditioned medium from J774M-KDR inhibited CD8^+^ T cell proliferation (Supplemental Figure 3, C and D). Overall, results using BM-myeloid cells from *Csf1r*-*Cre*^+^ *Flk-1^fl/fl^* mice and VEGFR2-overexpressing J774M cells demonstrate that elevated VEGFR2 expression on myeloid cells in TB animals is associated with their immunosuppressive phenotype and T cell–suppressive function.

### VEGF-VEGFR2–specific blockade reduces the immunosuppressive function of tumor-infiltrating myeloid cells.

**Figure 3:**
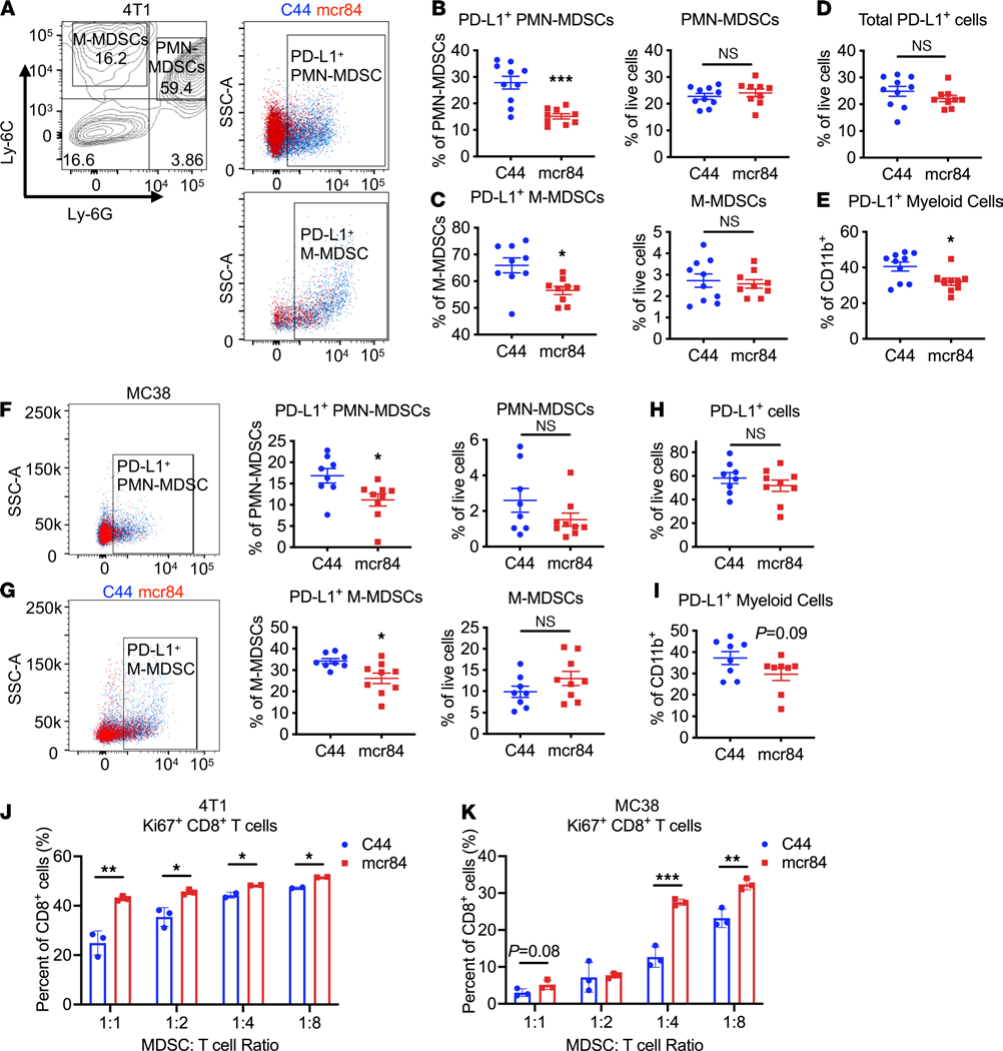
VEGF blockade by mcr84 decreases PD-L1 expression on myeloid cells. (**A**) Flow cytometry gating strategy for PMN-MDSCs and M-MDSCs and representative flow cytometry analysis of PD-L1 expression on gated PMN-MDSCs and M-MDSCs. (**B**–**E**) Flow cytometry analysis of the indicated cell types in 4T1 tumors treated as indicated. Each dot indicates 1 tumor. Expression of PD-L1 on PMN-MDSCs (**B**), M-MDSCs (**C**), and total CD11b^+^ myeloid cells (**E**) as well as total numbers of MDSCs (**B** and **C**) and PD-L1^+^ cells (**D**) were evaluated. (**F**–**I**) Flow cytometry analysis of the indicated cell types in MC38 tumors. The left panels in (**F**) and (**G**) show representative flow cytometry analysis of PD-L1 expression on gated PMN-MDSCs and M-MDSCs. Data are displayed as mean ± SEM with *n* = 9 to 10 per group analyzed. *, *P* < 0.05; ***, *P* < 0.001 vs. control, by Welch’s *t* test. (**J** and **K**) Sorted tumor-infiltrating MDSCs from C44- or mcr84-treated 4T1 (**J**) or MC38 (**K**) TB mice were cocultured with CD8^+^ T cells isolated from splenocytes of wild-type C57BL/6 mice at ratios indicated. After 72 hours, Ki67 expression was evaluated by flow cytometry. Data are displayed as mean ± SEM with *n* = 2 to 3 per group analyzed. *, *P* < 0.05; **, *P* < 0.005; ***, *P* < 0.001, by Welch’s *t* test.

Given the observation that the expression of VEGFR2 on myeloid cells results in an immunosuppressive phenotype in vitro (Figure 2, C–H), we analyzed how VEGF-VEGFR2–specific blockade affects the phenotype of infiltrating myeloid cells in 4T1 tumors. By performing flow cytometry, we identified that consistent with our in vitro assay (Figure 2, C and G), the expression of PD-L1 on 2 populations of MDSCs (Ly-6G^+^Ly-6C^dim^ PMN-MDSCs and Ly-6G^–^Ly-6C^+^ M-MDSCs) in the tumors was significantly downregulated by treatment with mcr84 (Figure 3, A–C). Importantly, we observed the decrease of PD-L1 was myeloid cell specific, since we did not find a decrease of PD-L1 expression in non–myeloid cell populations (Figure 3D). We investigated PD-L1 expression in other myeloid cell types and found that downregulation of PD-L1 was a general phenomenon in myeloid cells, including CD11b^+^ cells (Figure 3E), Ly-6C^+^ cells, Ly-6G^hi^ cells, total MDSCs, and neutrophils (Supplemental Figure 4A). However, PD-L1 expression on CD31^+^ endothelial cells and EpCAM^+^ tumor cells remained unchanged, while there was a trend of decrease in CD45^+^ cells (Supplemental Figure 4B). Interestingly, we found that mcr84 treatment reduced PD-L1 expression on splenic myeloid cells (Supplemental Figure 4C), indicating a systemic effect of VEGF blockade on PD-L1 expression. Results in the 4T1 model were generally recapitulated in MC38 (Figure 3, F–I) and E0771 tumors (Supplemental Figure 4, D–G). Similarly, we did not detect PD-L1 downregulation on endothelial cells or tumor cells in the MC38 model (Supplemental Figure 4H). However, in the MC38 model, mcr84 treatment induced a significant decrease of PD-L1 on CD45^+^ cells, which was mainly due to the myeloid component (Supplemental Figure 4H). In addition, we found mcr84 treatment suppressed MDSC accumulation in E0771 tumors (Supplemental Figure 4, D and E). To evaluate if the downregulated PD-L1 expression contributes to reversed immunosuppressive phenotype of tumor-infiltrating MDSCs, we sorted Gr-1^+^Ly-6G^+^ MDSCs from C44/mcr84-treated tumors using an MDSC isolation kit and cocultured with anti-CD3/CD28-stimulated wild-type CD8^+^ T cells at different ratios. The purity of sorted MDSCs was confirmed by flow cytometry (Supplemental Figure 4I). Using cells from 4T1 and MC38 models, MDSCs isolated from mcr84-treated tumors demonstrated less suppressive capacity with higher percentage of Ki67^+^ proliferating CD8^+^ T cells in coculture (Figure 3, J and K), indicating VEGF blockade by mcr84 resulted in less suppressive tumor-infiltrating MDSCs.

We also analyzed the phenotype of tumor-infiltrating myeloid cells by IHC. We found that mcr84 limited macrophage infiltration and altered macrophage phenotype toward iNOS^+^ immunostimulatory macrophages with decreased Arg-1 expression in 4T1 tumors (Supplemental Figure 5A). As expected, we found that tumor cell conditioned media induced high Arg-1 expression on BM-myeloid cells from NTB mice, a response that was not sensitive to VEGF blockade (Supplemental Figure 5B). However, BM-MQs differentiated from 4T1 TB animals that were treated with mcr84 demonstrated decreased Arg-1 expression and lower Arg-1^+^/iNOS^+^ ratio compared with TB animals treated with an isotype control antibody (Supplemental Figure 5C). These findings indicate that VEGF inhibition has an effect on BM differentiation where it contributes to macrophage polarization. These results were recapitulated in TAMs from MC38-bearing mice (Supplemental Figure 5D). Furthermore, in MC38 tumors, mcr84 increased MHCII expression on conventional DCs, which are the professional antigen-presenting cells (32), suggesting VEGF blockade enhances the maturation and antigen-presenting capacity of DCs (Supplemental Figure 5E). In summary, these in vivo data support our in vitro observations and suggest that VEGF drives an immunosuppressive phenotype in tumor-specific VEGFR2^+^ myeloid cells and that blockade of VEGF activation of VEGFR2 on myeloid cells can reduce myeloid immunosuppressive function.

### VEGF directly upregulates PD-L1 expression on myeloid cells through VEGFR2.

**Figure 4:**
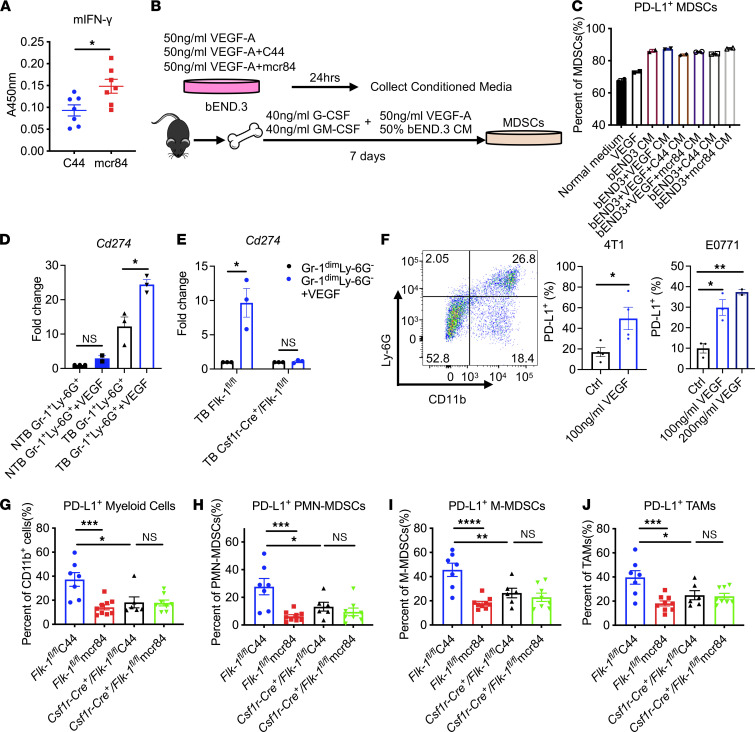
VEGF directly upregulates PD-L1 expression on myeloid cells through VEGFR2. (**A**) Intratumor IFN-γ level was analyzed from whole tumor lysates with indicated treatments by ELISA. Data are displayed as mean ± SEM with *n* = 7/group analyzed. *, *P* < 0.05 vs. control, by Welch’s *t* test. (**B** and **C**) bEnd.3 endothelial cells were pretreated with VEGF with/without C44 or mcr84 for 24 hours. Conditioned media (CM) were harvested, and BM from C57BL/6 mice was differentiated into MDSCs as shown in the schematics (**B**). On day 7, PD-L1 expression on MDSCs was analyzed by flow cytometry as shown (**C**). Data are displayed as mean ± SD with 2 independent experiments. (**D**) Gr-1^+^Ly-6G^+^ MDSCs were sorted from splenocytes of NTB mice and E0771 TB mice. (**E**) Gr-1^dim^Ly-6G^–^ M-MDSCs were sorted from splenocytes of *Flk-1^fl/fl^* and *Csf1r-Cre^+^ Flk-1^fl/fl^* TB mice. PD-L1 expression after VEGF (100 ng/mL) stimulation for 24 hours was evaluated by quantitative PCR. Three independent experiments using duplicate samples were performed. Data are displayed as fold change normalized to NTB mice or control (mean ± SEM). *, *P* < 0.05, by Welch’s *t* test. (**F**) CD11b^+^Ly-6G^+^ MDSCs were sorted from 4T1 and E0771 digested tumors (5–6 tumors pooled in each model) and were stimulated with VEGF (100 ng/mL or 200 ng/mL) for 48 hours. PD-L1 expression was analyzed by flow cytometry. Three to four independent experiments were performed (mean ± SEM). *, *P* < 0.05; **, *P* < 0.005, by Welch’s *t* test in 4T1 model or ANOVA with Tukey’s MCT in E0771 model. (**G**–**J**) F246-6 tumors grown in *Flk-1^fl/fl^* or *Csf1r-Cre^+^* *Flk-1^fl/fl^* mice were analyzed by flow cytometry for PD-L1 expression on indicated myeloid cells. Data are displayed as mean ± SEM with *n* = 6–9/group analyzed. *, *P* < 0.05; **, *P* < 0.005; ***, *P* < 0.001; ****, *P* < 0.0001 by ANOVA with Tukey’s MCT.

Since we had observed a consistent decrease of PD-L1 expression on myeloid cells across different syngeneic animal models after treatment with mcr84, we investigated whether this was a direct or indirect consequence. First, we analyzed the level of IFN-γ, a major inducer of PD-L1, in lysates from control and mcr84-treated tumors. Surprisingly, we found that the level of intratumor IFN-γ was elevated after mcr84 treatment (Figure 4A), highlighting that the decrease of PD-L1 on tumor-associated myeloid cells was not due to a reduction of IFN-γ in the tumor microenvironment. Therefore, we directly stimulated BM-myeloid cells from NTB mice with IFN-γ and VEGF with/without mcr84. As expected, IFN-γ alone elevated PD-L1 expression; however, VEGF with/without mcr84 did not affect PD-L1 expression (Supplemental Figure 5, F and G). These results indicate that the downregulation of PD-L1 by mcr84 is independent of IFN-γ. Given the fact that anti-VEGF therapy disrupted tumor blood vessels, we evaluated whether the modulation of VEGFR2 signaling in endothelial cells could alter the endothelial cell secretome, thus changing PD-L1 expression on myeloid cells. We added conditioned media from mouse bEnd.3 endothelial cells stimulated with VEGF with/without mcr84 to the differentiation media of BM-MDSCs (Figure 4B). PD-L1 level on BM-MDSCs was then analyzed by flow cytometry. Although conditioned media alone slightly induced PD-L1 expression on BM-MDSCs, the expression level remained similar regardless of activation or inhibition of VEGFR2 signaling on endothelial cells (Figure 4C).

To determine if VEGF/VEGFR2 signaling on myeloid cells directly enhances PD-L1 expression, Gr-1^+^Ly-6G^+^ MDSCs were sorted from splenocytes of NTB and TB mice and treated with VEGF for 24 hours. We found that PD-L1 expression level was elevated by VEGF on splenic MDSCs from TB mice compared with MDSCs from NTB mice (Figure 4D). To confirm the effect of VEGF, similar experiments were performed using splenic Gr-1^dim^Ly-6G^–^ M-MDSCs isolated from *Flk-1^fl/fl^* and *Csf1r*-*Cre*^+^ *Flk-1^fl/fl^* TB mice. Consistent with Gr-1^+^Ly-6G^+^ MDSCs, we found PD-L1 expression was stimulated by VEGF in MDSCs from *Flk-1^fl/fl^* mice; however, VEGF treatment did not change PD-L1 expression in splenic MDSCs from *Csf1r*-*Cre*^+^ *Flk-1^fl/fl^* mice (Figure 4E). In addition, in CD11b^+^Ly-6G^+^ MDSCs sorted from tumors, stimulation with VEGF led to a substantial increase of PD-L1 expression in both 4T1 and E0771 models (Figure 4F).

To further confirm the direct effect of mcr84 on myeloid cell PD-L1 expression in vivo, we implanted F246-6 breast cancer cell line in *Flk-1^fl/fl^* or *Csf1r*-*Cre*^+^ *Flk-1^fl/fl^* mice. Mice with established tumors were randomized to receive control IgG or mcr84. First, we found that consistent with earlier results (Figure 1, A–C), mcr84 alone slowed tumor growth in *Flk-1^fl/fl^* control animals; however, ablation of *Vegfr2* on myeloid cells abrogated the efficacy of mcr84 (Supplemental Figure 6A). In addition, we found that the effect of mcr84 on reducing total vessel density was also lost in the absence of *Vegfr2* on myeloid cells (Supplemental Figure 6B). These data demonstrate that VEGFR2^+^ myeloid cells are important in the efficacy of anti-VEGF therapy. We also analyzed macrophage infiltration and phenotype by IHC and flow cytometry. We found that loss of *Vegfr2* on myeloid cells resulted in reduced macrophage tumor infiltration similar to mcr84 treatment while in the absence of *Vegfr2*, mcr84 did not reduce macrophage infiltration (Supplemental Figure 6, C and D). We also observed decreased Arg-1 expression on macrophages and PMN-MDSCs with ablation of *Vegfr2* on myeloid cells (Supplemental Figure 6E). We then assessed PD-L1 expression on myeloid cells by flow cytometry. Consistently, in multiple myeloid cell types, including total CD11b^+^ myeloid cells, PMN-MDSCs, M-MDSCs, and macrophages, mcr84 treatment significantly downregulated PD-L1 expression regardless of the relative frequency of the myeloid cell population in control mice (Figure 4, G–J). In contrast, *Csf1r*-*Cre*^+^ *Flk-1^fl/fl^* mice had reduced levels of PD-L1 on myeloid cells compared with control mice, and VEGF blockade by mcr84 did not further reduce PD-L1 expression (Figure 4, G–J). These findings support our in vitro data (Figure 4, D–F) and demonstrate that the downregulation of PD-L1 on myeloid cells by VEGF blockade is a direct effect of inhibiting the VEGF/VEGFR2 signaling on myeloid cells.

Moreover, we analyzed the phenotype of tumor-infiltrating T cells in *Flk-1^fl/fl^* and *Csf1r*-*Cre*^+^ *Flk-1^fl/fl^* TB mice. Although we found that loss of *Vegfr2* resulted in downregulation of expression of immune checkpoint inhibitory receptors PD-1 and CTLA-4 on CD8^+^ T cells (Supplemental Figure 6F), the total abundance of T cells and PD-1^–^Ki67^+^ effector T cells was unchanged between these 2 groups (Supplemental Figure 6, G and H). Functionally, splenocytes from *Csf1r*-*Cre*^+^ *Flk-1^fl/fl^* TB mice have similar cytotoxic activity as those from *Flk-1^fl/fl^* mice when cocultured with parental F246-6 cells (Supplemental Figure 6I).

### VEGF blockade by mcr84 promotes perivascular accumulation of T cells and stimulates T cell activation.

**Figure 5:**
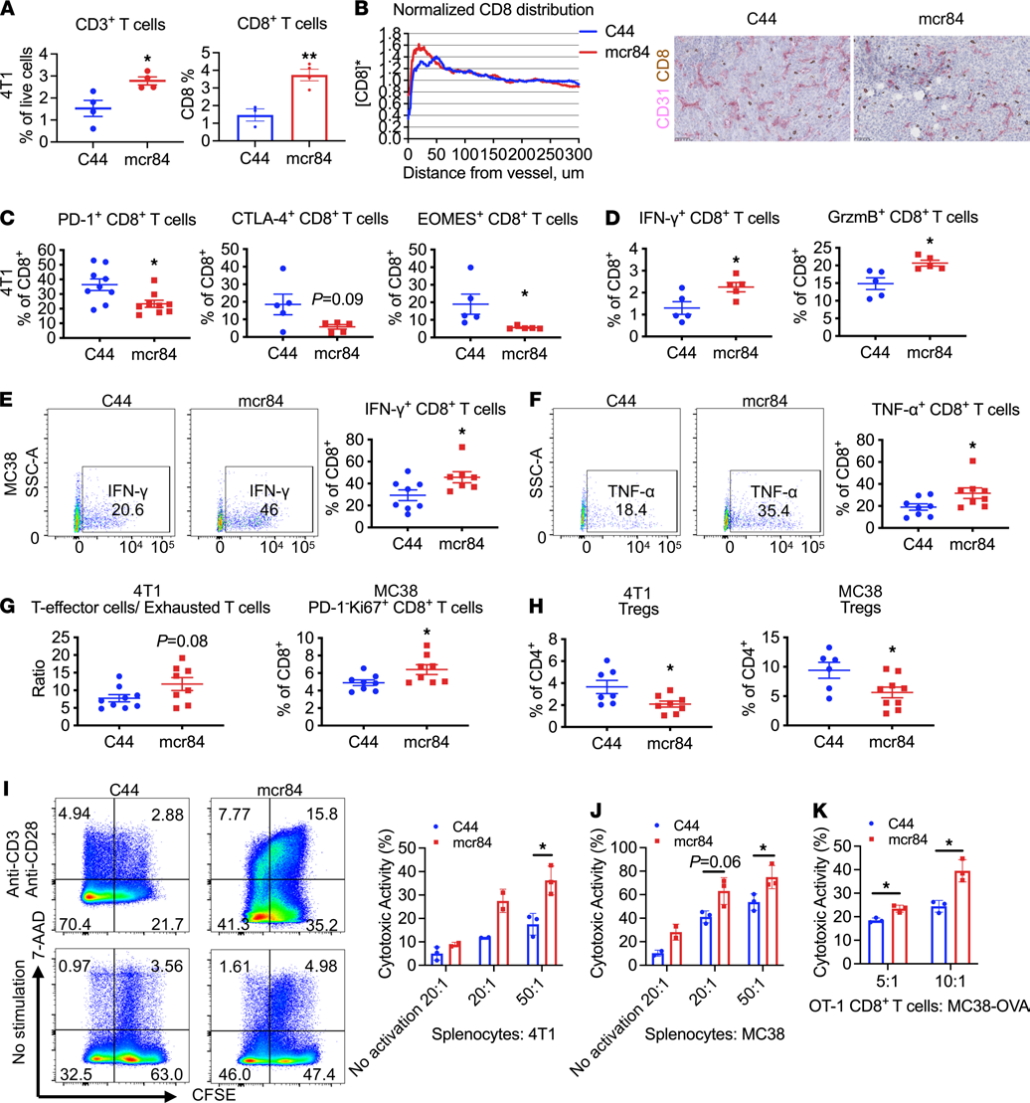
VEGF blockade by mcr84 promotes perivascular accumulation of T cells and stimulates T cell activation. (**A**) Flow cytometry analysis or IHC of the indicated cell types in 4T1 tumors. (**B**) Normalized distribution of CD8^+^ T cells around CD31^+^ blood vessels in 4T1 tumors (*n* = 5/group). Representative images of CD8 (brown) and CD31 (red) staining in 4T1 tumors are shown. Scale bar: 50 μm. (**C** and **D**) Flow cytometry analysis of PD-1, CTLA-4, EOMES, intracellular IFN-γ, and granzyme B on CD8^+^ T cells in 4T1 tumors treated as indicated. (**E** and **F**) Flow cytometry analysis of intracellular IFN-γ (**E**) and TNF-α (**F**) expression on CD8^+^ T cells in MC38 tumors treated as indicated. The left panels (**E** and **F**) show representative flow cytometry analysis of indicated cytokines in gated CD8^+^ T cells. (**G** and **H**) Flow cytometry analysis of T effector cells/exhausted T cells (**G**) and Tregs (**H**) in 4T1 and MC38 tumors treated as indicated. T effector cells were characterized as PD-1^–^Ki67^+^CD8^+^ T cells. Exhausted T cells were characterized as CTLA-4^+^PD-1^+^CD8^+^ T cells. Tregs were characterized as CD25^+^FoxP3^+^CD4^+^ T cells. Each dot indicates 1 tumor. Data are displayed as mean ± SEM with 5 to 9 animals per group. (**I**–**K**) An in vitro cell cytotoxicity assay was performed following the instructions of the basic cytotoxicity assay kit. Splenocytes from animals treated as indicated, or splenic CD8^+^ T cells from OT-1 MC38 TB mice were cocultured with CFSE prelabeled 4T1, MC38, or MC38-OVA cells at different ratios for 72 or 48 hours. Dead cells were labeled with 7-AAD. Samples were analyzed by flow cytometry. Representative images of gating strategy of 4T1 coculture are shown (**I**). Cytotoxicity percentages were calculated in (**I**) (4T1), (**J**) (MC38), and (**K**) (MC38-OVA). *n* = 2 to 3/group. *, *P* < 0.05; **, *P* < 0.005 vs. control, by Welch’s *t* test.

Because VEGFR2-specific blockade by mcr84 resulted in tumor vasculature that favored immune cell trafficking and reduced the immunosuppressive phenotype of myeloid cells, we analyzed T cell infiltration and activation in tumors after treatment with mcr84. Flow cytometry and IHC showed an increase of CD3^+^ and CD8^+^ T cells 1 week after the initiation of mcr84 therapy in 4T1 tumors (Figure 5A). In addition, distribution analysis of CD8^+^ T cells by IHC indicated that VEGF blockade promoted the perivascular accumulation of T cells in 4T1 tumors (Figure 5B). These data support that VEGF blockade reduces the vascular immune barrier (33) and results in elevated T cell tumor infiltration. Moreover, we performed IHC analysis of liver metastasis from patients with colorectal cancer receiving chemotherapy alone or chemotherapy combined with Avastin (bevacizumab) and found that VEGF blockade led to increased CD3^+^ and CD8^+^ T cell infiltration into metastatic sites (Supplemental Figure 7A). Consistent with preclinical data, Avastin treatment in patients with colorectal cancer resulted in decreased recruitment of macrophages to liver metastases and downregulated PD-L1 expression on macrophages (Supplemental Figure 7, B and C).

To evaluate whether 4T1 tumor-infiltrating T cells were functionally active, we analyzed the expression of immune checkpoint inhibitory receptors PD-1 and CTLA-4, T-box transcription factor EOMES, and cytokine production of intratumor CD8^+^ T cells by flow cytometry. We found that mcr84 treatment markedly decreased expression of PD-1, CTLA-4, and EOMES on CD8^+^ T cells, suggesting a reduced exhaustion status of cytotoxic T lymphocytes (CTLs; Figure 5C). Meanwhile, mcr84 significantly increased the proportion of CD8^+^ T cells expressing IFN-γ and granzyme B (Figure 5D). Similarly, CD8^+^ T cells in mcr84-treated MC38 tumors also exhibited an activated phenotype with elevated expression level of IFN-γ and TNF-α (Figure 5, E and F). Additionally, the ratio of T effector cells to exhausted T cells was increased after VEGF blockade in 4T1 and MC38 tumors (Figure 5G). Furthermore, mcr84 inhibited Treg infiltration in 4T1 and MC38 tumors (Figure 5H) while elevating CTLA-4 expression on Tregs (Supplemental Figure 7, D and E). These data suggest that the function of cytotoxic CD8^+^ T cells was enhanced by VEGF blockade.

We then performed an in vitro cell cytotoxicity assay to evaluate the activity of CTLs from 4T1 and MC38 TB mice after treatment with mcr84 or control IgG. Splenocytes from mAb-treated mice were stimulated with plate-bound anti-CD3 and soluble anti-CD28 antibodies and cocultured with CFSE-labeled 4T1 or MC38 tumor cells at different ratios. After 72 hours of coculture, dead cells were labeled by 7-AAD, and cells were analyzed by flow cytometry. Splenocytes from mcr84-treated mice had a higher level of cytotoxic activity in both models (Figure 5, I and J), suggesting that mcr84 treatment facilitates priming of CTLs. This phenotype was replicated by coculturing sorted CD8^+^ T cells from splenocytes of MC38 TB mice with parental MC38 tumor cells (Supplemental Figure 7F). To demonstrate if the cytotoxic activity of splenocytes is antigen dependent, we implanted MC38 tumor cells subcutaneously into OT-1 mice and treated the mice with C44 or mcr84 after tumors were established. MC38 tumor growth was faster in OT-1 mice since T cells were designed to recognize a certain ovalbumin (OVA) peptide (Supplemental Figure 7G). CD8^+^ T cells were isolated from splenocytes of mAb-treated mice and cocultured with CFSE-labeled MC38-OVA tumor cells or MC38 parental cells at different ratios. Dead cells were then analyzed after 48 hours of coculture. We found the cytotoxic capacity of CD8^+^ T cells sorted from mcr84-treated mice was higher than those from C44-treated mice in the coculture of MC38-OVA cells (Figure 5K), indicating antigen-specific cytotoxic activity. However, no significant difference was observed between CD8^+^ T cells of different treatments in the coculture of MC38 parental cells, and in general, CD8^+^ T cells exhibited low cytotoxic activity against MC38 cells (Supplemental Figure 7H). Consistent with the in vitro cell cytotoxicity assay, histological analysis of in vivo 4T1 and MC38 tumors demonstrated that mcr84-treated tumors showed elevated cleaved caspase-3 (CC3) in the 4T1 model, indicating increased tumor cell death and decreased Ki67 expression in the 4T1 and MC38 models (Supplemental Figure 7, I and J).

Taken together, our data suggest that inhibition of VEGF/VEGFR2 signaling stimulates the activation of tumor-infiltrating T cells and facilitates the functional activity of CTLs in TB animals, which might be attributed to the inhibition of VEGFR2^+^ immunosuppressive myeloid cells.

### VEGF blockade enhances the efficacy of CTLA-4 blockade in tumors.

**Figure 6:**
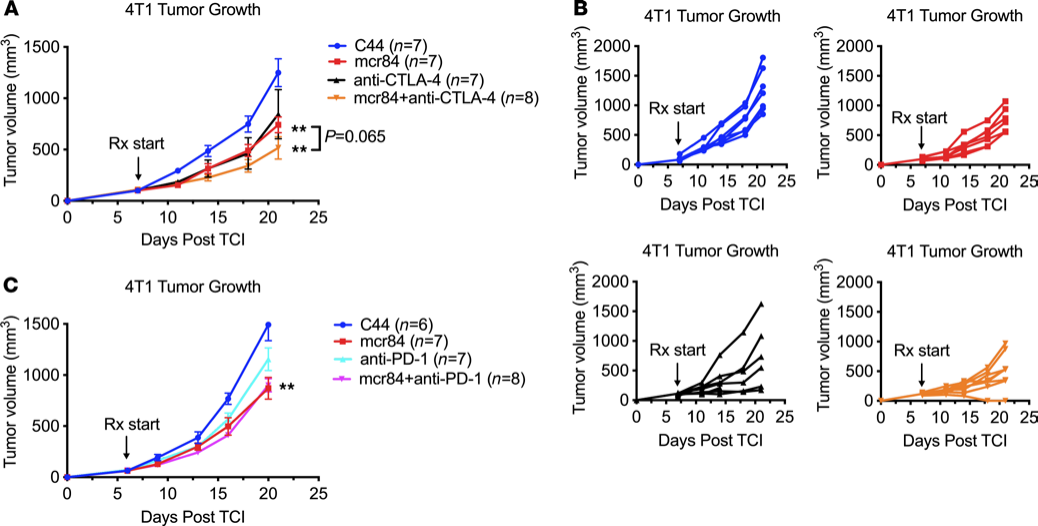
CTLA-4 blockade enhances the antitumor activity of mcr84. (**A**) A total of 1 × 10^5^ 4T1 cells (*n* = 7–8/group) were injected orthotopically into 8-week-old BALB/c mice. Mice with established tumors (50–150 mm^3^) were treated with control antibody (C44, 250 μg/dose, twice per week), mcr84 (250 μg/dose, twice per week), anti–CTLA-4 antibody (clone: 9d9, 100 μg/dose, every 3 days) or mcr84 and anti–CTLA-4. Mice were monitored daily and tumor volume was measured twice per week. All mice were sacrificed when tumor volume in the control group reached 2000 mm^3^. Tumor growth was analyzed. Data are displayed with mean ± SEM. **, *P* < 0.005 vs. control C44, by Welch’s *t* test. (**B**) Growth curves of individual tumors. Arrows indicate start of treatments (day 7). Colors of labeling correspond to legends in **A**. (**C**) Combination efficacy of VEGF and PD-1 blockade in 4T1 syngeneic model. Experiment was performed similarly as described in **A**. Anti–PD-1 antibody (clone: RMP14-1, 100 μg/dose, i.p.) was dosed twice per week. Data are displayed with mean ± SEM. **, *P* < 0.005 vs. control C44, by ANOVA with Tukey’s MCT.

Given the fact that treatment with mcr84 results in the downregulation of PD-L1 on myeloid cells but upregulation of CTLA-4 on Tregs in multiple models (Supplemental Figure 7, D and E), we sought to evaluate the therapeutic efficacy of combining VEGF blockade with PD-1 or CTLA-4 inhibition in 4T1 tumors. We found that mcr84 significantly enhanced the efficacy of CTLA-4 but not PD-1 blockade (Figure 6, A–C). These results are consistent with the data that PD-L1 on myeloid cells was downregulated by VEGF inhibition.

**Figure 7:**
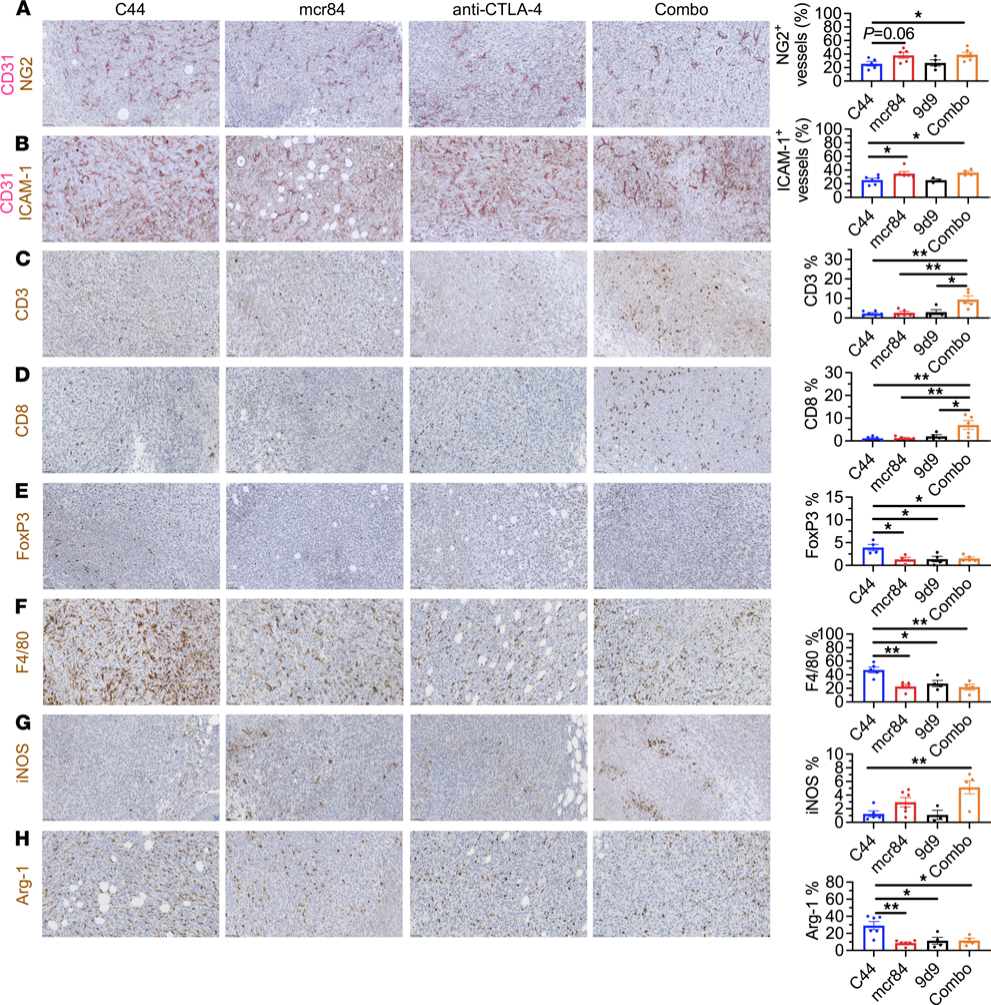
VEGF blockade in combination with anti–CTLA-4 therapy increases T cell infiltration and polarizes macrophages to an immunostimulatory phenotype. FFPE 4T1 tumors were assessed for CD31 and NG2 (**A**); CD31 and ICAM-1 (**B**); T cell markers CD3 (**C**), CD8 (**D**), and FoxP3 (**E**); as well as macrophage markers F4/80 (**F**), iNOS (**G**), and Arg-1 (**H**). Slides were scanned and images were analyzed using NIS Elements (Nikon) and Fiji software. Representative images are shown with CD31 in red and other markers in brown. Scale bar, 50 μm. Quantification is shown to the right. Data are displayed as mean ± SEM (*n* = 4–6/group). *, *P* < 0.05; **, *P* < 0.005, by ANOVA with Tukey’s MCT.

Moreover, we performed IHC and found that combination of mcr84 and anti–CTLA-4 reduced microvessel density, increased pericyte coverage of blood vessels, increased the expression of ICAM-1, and importantly, increased CD3^+^ T cell and CD8^+^ cytotoxic T cell infiltration into tumors (Figure 7, A–D). At the same time, FoxP3^+^ Treg and macrophage infiltration were significantly decreased by mcr84 and the combination therapy (Figure 7, E and F). Consistent with our earlier results, mcr84 in combination with anti–CTLA-4 also decreased immunosuppressive Arg-1^+^ and increased immunostimulatory iNOS^+^ macrophages (Figure 7, G and H). These results suggest that VEGF blockade potentiates the efficacy of anti–CTLA-4 therapy by increasing T cell infiltration and polarizing macrophages to an immunostimulatory phenotype. Collectively, our findings provide a rationale for combining anti-VEGF therapy with ICIs and indicate the potential possibility of combining VEGFR2 inhibition with immune checkpoint blockade other than anti–PD-1/PD-L1.

## Discussion

Compelling studies have demonstrated the benefit of antiangiogenic therapy in combination with ICIs in preclinical and clinical settings (4). In this study, we identified and characterized the function of a population of myeloid cells expressing VEGFR2 specifically in TB animals and investigated the mechanism(s) of how VEGF blockade promotes an immunostimulatory tumor microenvironment, including effects on tumor endothelium, tumor-associated myeloid cells, and TILs. Selective inhibition of VEGF activation of VEGFR2 with mcr84 increased the expression of adhesion molecules while decreasing expression of FasL on tumor endothelial cells reduced the expression of PD-L1 on myeloid cells, reversed the immunosuppressive phenotype of tumor-infiltrating myeloid cells, and reduced the expression of inhibitory immune checkpoint molecules on TILs. These changes resulted in increased T cell infiltration into tumors and increased TILs’ antitumor activity.

Mechanistically, we found that the decreased PD-L1 expression on tumor-infiltrating myeloid cells was due to direct inhibition of VEGFR2 on myeloid cells in TB animals. We have previously demonstrated that a subset of TAMs express VEGFR2, which mediates VEGF-induced infiltration into tumors (12). VEGFR2 was also found to be expressed on BM-derived plasmacytoid DCs and responsible for production of type I IFN and cell proliferation (34). A recent study has demonstrated that in murine gliomas and patients with glioma, elevated VEGFR2 expression on myeloid cells is associated with malignancy and high disease grade. Deficiency of VEGFR2 in BM-derived cells restrains the differentiation of myeloid lineages and proangiogenic function (35). Here, we showed that VEGFR2 was selectively upregulated on myeloid cells in TB animals, and the expression directly contributed to the myeloid cell immunosuppressive phenotype and elevated PD-L1 expression in response to VEGF stimulation. Thus, blocking VEGF binding to VEGFR2 in vivo results in consistent and substantial downregulation of PD-L1 expression and less T cell–suppressive capacity of myeloid cells, especially MDSCs.

Given the fact that VEGFR2 is expressed by multiple types of immune cells, and tumor cells we have used have limited to no VEGFR2 expression, the efficacy of VEGFR2 inhibition we have shown in breast and colon cancer models is due to dual targeting of tumor endothelium and immune cells. To specify the contribution of myeloid cell VEGFR2, we exploited a genetic mouse model that does not express VEGFR2 on myeloid cells (*Csf1r^Cre+^ Flk-1^fl/fl^*). Our data reveal that specific deletion of *Vegfr2* on myeloid cells led to reduced response to VEGF blockade in a syngeneic breast cancer model, highlighting the importance of VEGFR2^+^ myeloid cells for the efficacy of anti-VEGF therapy. How VEGFR2^+^ myeloid cells contribute to VEGF-induced angiogenesis is an active area of investigation. However, *Vegfr2*-specific single depletion on myeloid cells is not sufficient to restrain tumor progression at least in 1 MMTV-PyMT–derived syngeneic breast tumor model, since proliferative and cytotoxic capacities of T cells were not significantly improved.

Our findings that VEGF inhibition affected T cell infiltration, exhaustion, and activation are consistent with previous studies (18, 36). The regulation of inhibitory checkpoint molecule expression on CD8^+^ T cells by VEGF in tumors was attributed to activation of the VEGFR2/PLCγ/calcineurin/NFAT pathway (18). These observations have been expanded by recent studies that have shown VEGF-induced, T cell exhaustion–specific transcriptional programs are dependent on TOX in microsatellite stable colorectal cancers (37).

Recently, a global, open-label, phase III trial has shown that the VEGF-blocking mAb bevacizumab combined with anti–PD-L1 improves overall and progression-free survival outcomes compared with standard-of-care sorafenib in unresectable hepatocellular carcinoma (38, 39). Clinical studies are also investigating anti-VEGF strategies in combination with other immune therapies, including CTLA-4 blockade or CD40 agonism (40–42). Our preclinical data reveal that specific inhibition of VEGF binding to VEGFR2 led to downregulation of PD-L1 on myeloid cells. PD-L1 expressed on myeloid cells was shown previously to directly reduce T cell function, highlighting the contribution of myeloid cells to ICI efficacy (43). Given the importance of PD-L1 expression on host BM-derived cells and DCs for the response of PD-L1 blockade (43, 44), our data provide a molecular rationale for how anti-VEGF enhances the efficacy of ICIs and highlight that inhibition of VEGFR2 activation might especially improve the efficacy of blocking immune checkpoint molecules besides the PD-1/PD-L1 axis.

However, the mechanism of how and when myeloid cells upregulate VEGFR2 expression in tumor progression as well as the signaling pathway of VEGF regulating PD-L1 expression remain unclear. Our preliminary data have demonstrated that VEGFR2 expression on BM-MQs can be induced by lipopolysaccharide stimulation in vitro. Further experiments to investigate these questions are underway. Mice with a VEGFR2 reporter might be beneficial to identify cell types that express VEGFR2 in tumors and study the distribution and infiltration of this VEGFR2^+^ myeloid population. In addition, macrophage-specific RNA-Seq data from Raphael Nemenoff’s group (45) indicate that VEGFR2 expression is strongly correlated with angiopoietin-1 receptor (Tie2) expression in normal and Lewis lung carcinoma macrophages (data not shown), which suggests a relationship between VEGFR2^+^ myeloid cells and Tie2-expressing monocytes as well as myeloid cells expressing other endothelial cell markers (46).

## Methods

### Cell lines.

Murine breast cancer cell line, 4T1, murine colon adenocarcinoma cell line, MC38, and HEK293T were obtained from ATCC. E0771, a murine breast cancer cell line, was a gift from Philip Thorpe (UT Southwestern Medical Center, Dallas, Texas, USA). F246-6, an isogenic breast cancer cell derived from MMTV-PyMT mice (47), was a gift from Jeff Pollard (University of Edinburgh, Edinburgh, United Kingdom). J774M, an MDSC-like cell line, was a gift from Kebin Liu (Medical College of Georgia, Augusta, Georgia, USA). MC38-OVA cell line was a gift from Yang-Xin Fu (UT Southwestern). Cells were cultured in DMEM (Invitrogen) or RPMI (Invitrogen) containing 10% FBS, maintained at 37°C in a humidified incubator with 5% CO_2_ and 95% air, and confirmed to be pathogen free before use.

### Animal studies.

BALB/c and C57BL/6 mice were purchased from The Jackson Laboratory. *Csf1r*-*Cre*^+^ *Flk-1^fl/fl^* mice were generated by breeding *Flk-1^fl/fl^* mice (a gift from Masanori Hirashima, Niigata University, Tokyo, Japan) with *Csf1r^Cre^* mice from The Jackson Laboratory. OT-1 mice were a gift from Yang-Xin Fu (UT Southwestern). All animals were housed in a pathogen-free facility with 24-hour access to food and water. Mouse breast cancer cells, 4T1 (1 × 10^5^) or E0771 (1 × 10^5^), were injected orthotopically into 8-week-old female BALB/c or C57BL/6 mice, respectively. Mouse colorectal carcinoma cells, MC38 (1 × 10^5^) cells, were injected subcutaneously into 8-week-old C57BL/6 mice. F246-6 cells (5 × 10^5^) were injected orthotopically into *Flk-1^fl/fl^* and *Csf1r*-*Cre*^+^ *Flk-1^fl/fl^* mice on a pure FVB background. MC38-OVA (1 × 10^5^) cells were injected subcutaneously into 6-week-old OT-1 mice. Tumor volumes were measured twice weekly using a digital caliper, and volumes were calculated using the formula: V = (a × b^2^)/2, where a is the largest dimension and b is the smallest dimension. Mice were randomized for different treatments, and treatments started when tumors were established (50–150 mm^3^) and ended when the tumor volume from the control group reached 2000 mm^3^. Treatment groups consisted of C44 (i.p. 12.5 mg/kg [~250 μg], twice per week), mcr84 (i.p. 12.5 mg/kg [~250 μg], twice per week) (developed in the Brekken lab as described previously; ref. 2), anti–CTLA-4 (clone: 9d9, Bio X Cell; i.p. 5 mg/kg [~100 μg], every 3 days), anti–PD-1 (clone: RMP14-1, Bio X Cell; i.p. 5 mg/kg [~100 μg], twice per week), or anti–CTLA-4 or anti–PD-1 in combination with mcr84 at the indicated dose. All mice were sacrificed at the same time or at similar tumor sizes. Tissues were fixed in 10% formalin or snap frozen in liquid nitrogen for further studies or digested into single-cell suspension for flow cytometry.

### Histology and tissue analysis.

Formalin-fixed tissues were embedded in paraffin and cut into 5 μm sections. Sections were evaluated by H&E and immunohistochemical analysis following our previously reported protocol (48) using antibodies specific for CD31 (Cell Signaling Technology, 77699), NG2 (MilliporeSigma, ab5320), VCAM-1 (Cell Signaling Technology, 32653S), ICAM-1 (Abcam, ab179707), FasL (Santa Cruz Biotechnology, NOK-1), CC3 (Cell Signaling Technology, 9664), Ki67 (Abcam, ab15580), CD3 (Thermo Fisher Scientific, PA1-29547), CD8 (Cell Signaling Technology, 98941S), FoxP3 (R&D Systems, MAB8214), F4/80 (Cell Signaling Technology, 70076S), iNOS (Thermo Fisher Scientific, PA1-21054), Arg-1 (Cell Signaling Technology, 93668S), human CD3 (Thermo Fisher Scientific, PA1-29547), human CD8 (Abcam, ab93278), human CD206 (Cell Signaling Technology, 91992S), and human PD-L1 (Cell Signaling Technology, 13684S). Negative controls included omission of primary antibody. Color images were obtained with Hamamatsu Nanozoomer 2.0HT using NDPview2 software. Pictures were analyzed using NIS Elements (Nikon) and Fiji software. Quantification is shown as percentage of area fraction.

### Flow cytometry analysis.

Tumors were digested with a cocktail containing collagenase I (45 U/mL; Worthington), collagenase II (15 U/mL; Worthington), collagenase III (45 U/mL; Worthington), collagenase IV (45 U/mL; Worthington), elastase (0.075 U/mL; Worthington), hyaluronidase (30 U/mL; MilliporeSigma), and DNase type I (25 U/mL; MilliporeSigma) for 60 minutes at 37°C and passed through a 70 μm cell strainer (Falcon). Splenocytes were isolated from spleens and passed through a 70 μm cell strainer (Falcon). Suspensions were washed twice with PBS and stained with Ghost Viability Dye 510 (BD Biosciences) for 15 minutes. The cell suspensions were then washed and stained with antibodies detecting CD11b (BD Biosciences, 557657), Ly-6C (BD Biosciences, 562728), Ly-6G (BD Biosciences, 740953), F4/80 (Biolegend, 123132), CD274 (PD-L1, BD Biosciences, 563369), CD11c (BD Biosciences, 564079), I-A/I-E (BD Biosciences, 562009), CD3 (BD Biosciences, 553061), CD4 (BD Biosciences, 562891), CD8 (BD Biosciences, 563332), CD279 (PD-1, BD Biosciences, 563059), CD152 (CTLA-4, BD Biosciences, 565778), CD25 (IL-2 receptor α, BD Biosciences, 562694), CD31 (BD Biosciences, 553373), ICAM-1 (CD54, BD Biosciences, 565987), CD45 (BD Biosciences, 553080) and EpCAM (CD326, eBioscience, 17-5791-82) for 1 hour at 4°C. To assess cytokine secretion, tumor single-cell suspension was stimulated with PMA (50 ng/mL, MilliporeSigma)/ionomycin (1 μg/mL, MilliporeSigma)/Brefeldin A (10 μg/mL, BD Biosciences) for 4 hours before surface staining. Surface-stained cells were fixed, permeabilized, and stained for intracellular markers Arg-1 (R&D Systems, IC5868P), iNOS (Thermo Fisher Scientific, 17-5920-82), FoxP3 (BD Biosciences, 560401), Ki67 (BioLegend, 652404), VEGFR2 (Cell Signaling Technology, 9698S), IFN-γ (BD Biosciences, 564336), Granzyme B (eBioscience, 11-8898-82), TNF-α (eBioscience, 12-7321-82), and EOMES (eBioscience, 25-4875-82). Cells were analyzed using FACS LSRFortessa SORP (BD Biosciences), and analysis was performed using FlowJo, with the help of the Moody Foundation flow cytometry facility at UT Southwestern Medical Center.

### Coculture experiment and T cell proliferation assay.

The coculture cytotoxicity assay was performed following the instructions of the basic cytotoxicity assay kit (969, ImmunoChemistry Technologies). In brief, target cancer cells were incubated with CellTrace CFSE (5 μM) (ImmunoChemistry Technologies) for 20 minutes at room temperature, then washed with cell culture medium with FBS. Splenocytes from mice of different treatments underwent red blood cell lysis and then were stimulated with plate-bound anti-CD3 antibody (1 μg/mL, BioLegend) and soluble anti-CD28 antibody (2.5 μg/mL, BioLegend). Splenocytes and prelabeled cancer cells were then cocultured for 72 hours. In the coculture of splenocytes from OT-1 mice with MC38-OVA or MC38 cells, T cells were not stimulated by anti-CD3/CD28 antibodies and coculture was 48 hours. Cells were harvested and labeled with 7-AAD (ImmunoChemistry Technologies). Cell percentages were analyzed by flow cytometry. The cytotoxicity percentage was calculated using the formula (7-AAD and CFSE double-positive cells %/CFSE-positive cells %) × 100%.

CD8^+^ T cells from splenocytes of wild-type C57BL/6 mice were isolated using CD8a^+^ T Cell Isolation Kit according to manufacturer instructions (Miltenyi Biotec) and labeled with CellTrace CFSE (1 μM) (Invitrogen, C34554). BM-derived myeloid cells from different mice at day 6 or conditioned media from 48 hours’ culture of J774M cells were harvested and added to CD8^+^ T cells at different ratios. Percentage of proliferating CD8^+^ T cells after 72 hours was analyzed by CFSE signal or intracellular Ki67 staining with flow cytometry. CD8^+^ T cells stimulated with plate-bound anti-CD3 antibody (1 μg/mL, BioLegend) and soluble anti-CD28 antibody (2 μg/mL, BioLegend) were used as a positive control.

### ELISA.

For ELISA array, assay was performed with tumor lysates following the instructions of the mIFN-γ DuoSet ELISA kits (R&D Systems). In summary, 96-well plates were incubated with capture antibody overnight at room temperature, washed with wash buffer, blocked with reagent diluent for 1 hour, and washed again. Then, 100 μL of sample was added per well to the plates and incubated for 2 hours at room temperature. The plates were washed and 100 μL of detection antibody was added per well and incubated for 2 hours at room temperature. Then, the plates were further washed and 100 μL of substrate solution was added to each well and incubated for 20 minutes at room temperature. Afterward, 50 μL of stop solution per well was used to stop the reaction, and the absorbance at 450 nm was measured.

### Generation of BM-derived myeloid cells.

BM cells were obtained from NTB C57BL/6, BALB/c, and *Csf1r-Cre^+^ Flk-1^fl/fl^* mice and 4T1, E0771, MC38, and F246-6 TB mice using standard techniques (49). A total of 40 ng/mL GM-CSF and 40 ng/mL G-CSF were used to differentiate BM into MDSCs and 20% L929 conditioned medium was used for macrophages’ differentiation. L929 conditioned medium was generated in the lab as previously described (49). Fresh medium with GM-CSF/G-CSF or L929 condition medium was added on day 3. BM-MDSCs and BM-MQs were harvested on day 6 for quantitative PCR or flow cytometry analysis.

### Real-time quantitative PCR.

MDSCs sorted from splenocytes, BM- MDSCs, or BM-MQs harvested on day 6 were subjected to RNA extraction using QIAGEN RNeasy Mini Kit (catalog 74106). Then, cDNA was synthesized using a Bio-Rad iScript cDNA synthesis kit. The expression of *Vegfr1* (forward: 5′-CCAGAGAGGCAGAGTGGTTG-3′; reverse: 5′-GCTCCTCTCAGACTGCCTTG-3′), *Vegfr2* (forward: 5′-CGTTAAGCGGGCCAATGAAG-3′; reverse: 5′-GCTCATCCAAGGGCAATTCATC-3′), and *Cd274* (forward: 5′-GCATTATATTCACAGCCTGC-3′; reverse: 5′-CCCTTCAAAAGCTGGTCCTT-3′) was measured by SYBR Green–dependent (Bio-Rad) quantitative PCR. Three independent experiments were performed. Duplicates were run in each experiment.

### Isolation of MDSCs from splenocytes and tumors.

Splenocytes were isolated from spleens of NTB or TB animals, passed through a 70 μm cell strainer (Falcon), and subjected to red blood cell lysis. Isolation of MDSCs (Gr-1^+^Ly-6G^+^) or M-MDSCs (Gr-1^dim^Ly-6G^–^) was performed using the myeloid-derived suppressor cell isolation kit (130-094-538, Miltenyi Biotec). Tumor-infiltrating CD11b^+^Ly-6G^+^ MDSCs from digested tumor single-cell suspension were isolated by FACS. Recombinant mouse VEGF-A_165_ (100 ng/mL or 200 ng/mL, BioLegend) was used for stimulation.

### Human VEGFR2 overexpression.

pHAGE constructs were prepared from a single colony and used to generate lentivirus for J774M infection as previously described (50). pHAGE-KDR was a gift from Gordon Mills and Kenneth Scott (Addgene plasmid 116754). pHAGE_puro was a gift from Christopher Vakoc (Addgene plasmid 118692) and was used as control. HEK293T cells were plated 18 to 24 hours before transfection at an initial confluence of 70% to 90%. Lentivirus was generated in HEK293T cells by transfecting the pHAGE constructs and 2 packaging plasmids (psPAX2 and pMD2.G) with Lipofectamine 2000 (Thermo Fisher Scientific) for 24-hour transfection. Virus was harvested every 24 hours for 2 days and passed through a 0.45 μm filter. J774M cells were transduced by centrifugation at 800*g*, 37°C, for 1 hour in the presence of polybrene. After 24 hours, transduction was repeated for a total of 3 times. A total of 4 μg/mL puromycin was used for selection of the target cells. After selection, J774M-KDR and J774M-Ctrl cells were sorted into single cells in 96-well plates. VEGFR2 expression was evaluated by flow cytometry, and clones with high VEGFR2 level (clones A6 and F6) were chosen for further use.

### Statistics.

Data were analyzed using GraphPad software. Results are expressed as mean ± SEM or mean ± SD. Data were analyzed by Welch’s *t* test or by 1-way ANOVA with the Tukey’s test for multiple comparisons, and results were considered significant at *P* < 0.05. All in vitro experiments were performed with 2 to 4 biological replicates.

### Study approval.

Animal experiments in this study were performed in accordance with an animal protocol (2018-102540) approved by the Institutional Animal Care and Use Committee at UT Southwestern. Liver tissue biopsies from patients with colorectal cancer who received chemotherapy (FOLFOX or FOLFIRINOX) or chemotherapy combined with Avastin followed by surgery for metastatic site resection were obtained under a protocol approved by the UT Southwestern Institutional Review Board (STU 042015-049).

## Author contributions

RAB, YZ, and HH designed the studies; YZ, MC, and AZ conducted experiments and acquired data; YZ, HH, and RAB analyzed data; PG and SMK provided reagents/tissues; YZ wrote the manuscript; and HH and RAB edited the manuscript.

## Supplementary Material

Supplemental data

## Version 1 10/21/2021

In-Press Preview

## Version 2 12/08/2021

Electronic publication
