# Supplementary material for: VEGFR2 activity on myeloid cells mediates immune suppression in the tumor microenvironment
Source: JCI Insight. 2021 Dec 8;6(23):e150735. doi: 10.1172/jci.insight.150735 (PMC8675197; doi:10.1172/jci.insight.150735)
Supplement: Supplemental data [file jciinsight-6-150735-s147.pdf]

VEGFR2 activity on myeloid cells mediates immune suppression in the tumor microenvironment

Authors: Yuqing Zhang[1,2,3], Huocong Huang[1,2], Morgan Coleman[1,4], Arturas Ziemys[5], Purva Gopal[6], Syed M. Kazmi[7], Rolf A. Brekken[1,2,3,#]

Affiliations: [1]Hamon Center for Therapeutic Oncology Research, [2]Department of Surgery, [3]Cancer Biology Graduate Program, [4]Division of Pediatric Hematology and Oncology, [6]Department of Pathology, [7]Division of Hematology and Oncology, UT Southwestern, Dallas, TX
[5]Program of Mathematics in Medicine, Houston Methodist Research Institute, Houston, TX

Supplementary Figures and Legends

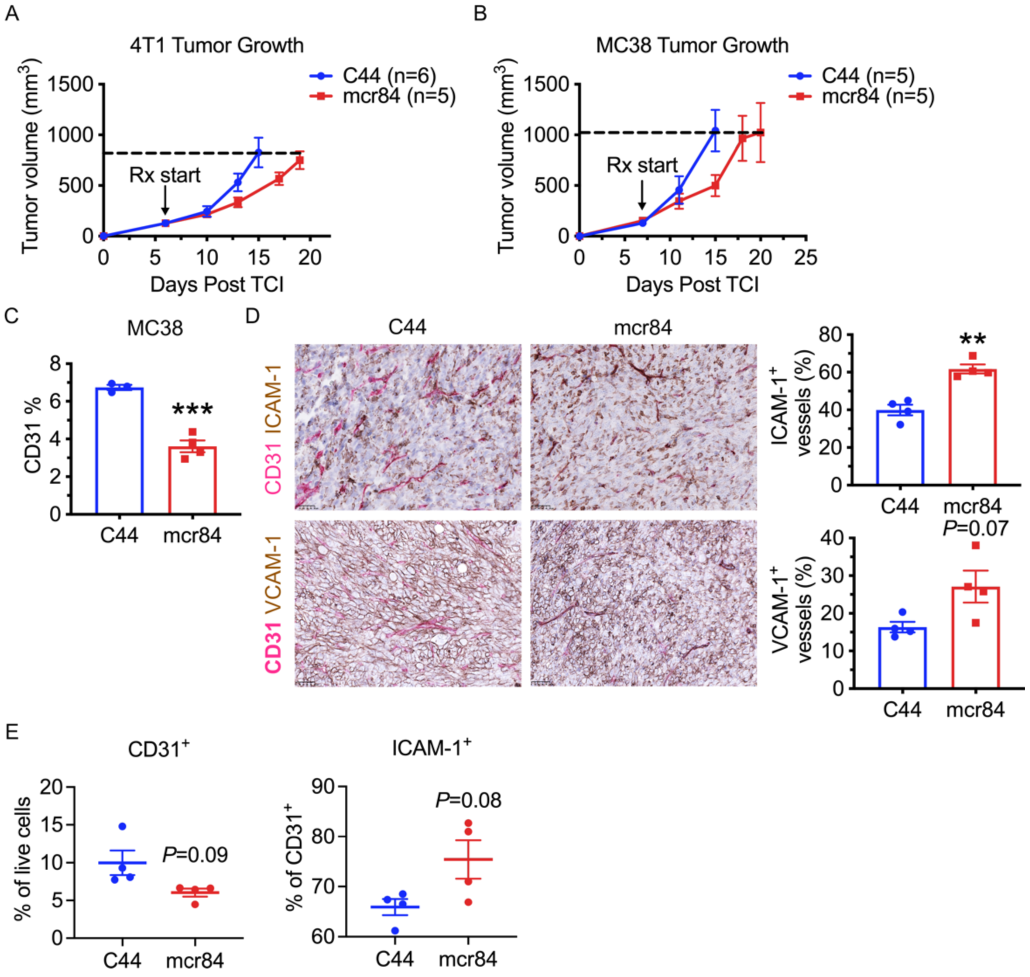

Supplementary Figure 1. mcr84 reduces microvessel density but increases adhesion molecule expression on blood vessels. (A) 1 × 10^5 4T1 cells (n=5-6/group) were injected orthotopically into 8-week-old BALB/c mice. (**B)** 1 × 10^5 MC38 cells were injected subcutaneously into 8-week-old C57BL/6 mice (n=5/group). Mice with established tumors (50–150 mm^3) were treated with control antibody (C44, 250 µg/dose, twice per week) or mcr84 (250 µg/dose, twice per week). Mice were monitored daily and tumor volume was measured twice per week. All mice were sacrificed at similar tumor sizes. Data are displayed as mean ± SEM**. (C-D)** Immunohistochemistry for CD31 and adhesion molecules (ICAM-1, VCAM-1) on FFPE MC38 tumors. Slides were scanned and images were analyzed using NIS Elements (Nikon) and Fiji software. Representative images are shown with CD31 in red and adhesion molecules in brown. Scale bar, 50 µm. Quantification is shown. Data are displayed as mean ± SEM (n=4/group). **, $P < 0.005$; ***, $P < 0.001$ vs. control by Welch's *t* test. (**E)** Flow cytometry analysis of endothelial cells (CD31) and endothelial expression of ICAM-1 in 4T1 tumors treated with mcr84 for one week. Each dot indicates one tumor. Data are displayed as mean ± SEM (n=4-5/group). *, $P < 0.05$, by Welch's *t* test.

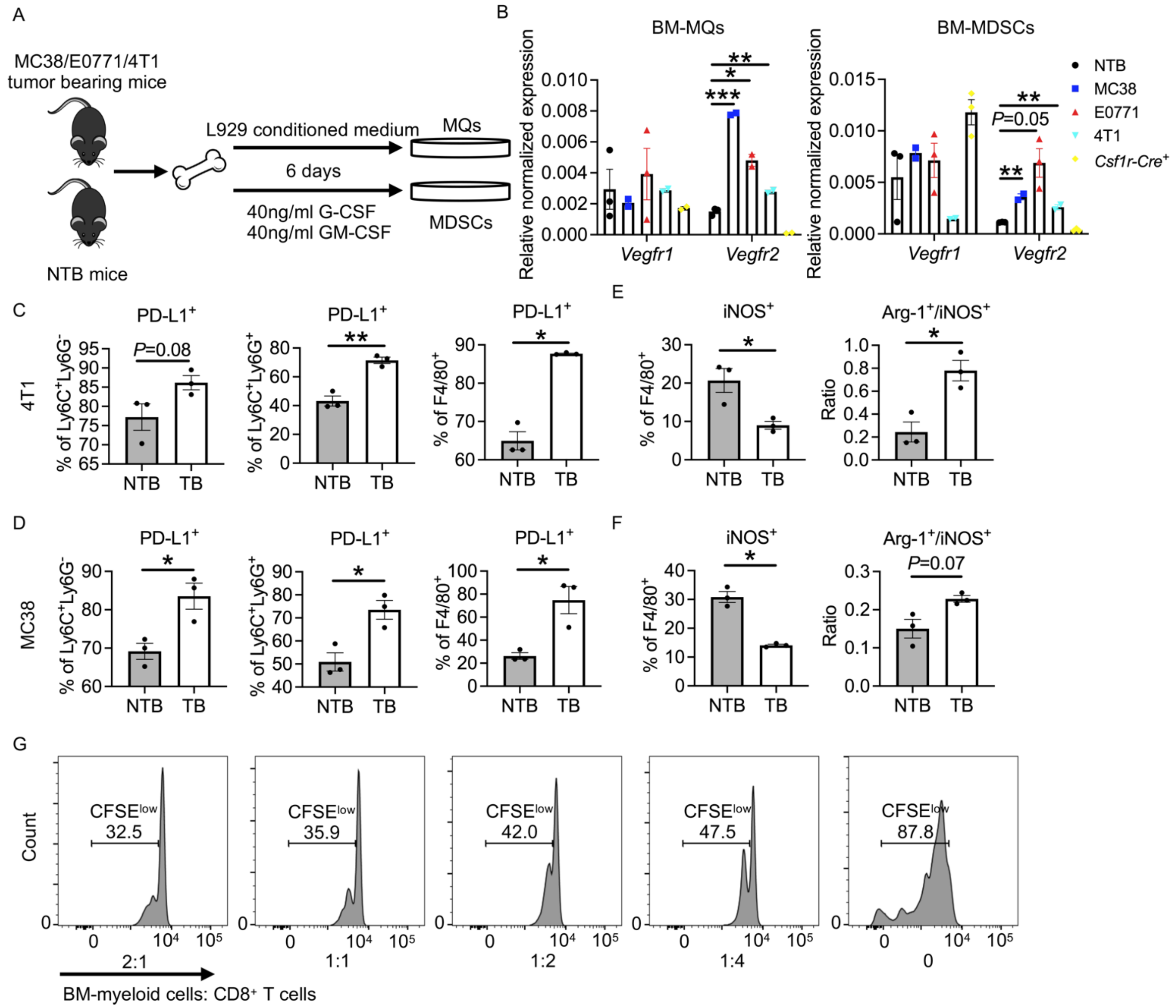

Supplementary Figure 2. Expression of VEGFR2 on myeloid cells is elevated specifically in tumor-bearing animals and is associated with an immunosuppressive myeloid phenotype. (**A-B)** Bone marrow (**BM**)-derived macrophages (**MQs**) and MDSCs from non-tumor bearing (**NTB**) animals, MC38, E0771 and 4T1 tumor-bearing **(TB)** animals and *Csf1r-Cre*$^{+}$ *Flk-1*$^{fl/fl}$ animals were analyzed for *Vegfr1* and *Vegfr2* expression. Schematic experimental design is shown in (A). *Vegfr1* and *Vegfr2* expression were evaluated by qPCR (B). Data are displayed as mean ± SEM with three independent experiments using duplicate samples. *, $P < 0.05$; **, $P < 0.005$; ***, $P < 0.001$ vs. NTB control by Welch's *t* test. (**C-F)** BM-derived myeloid cells from NTB mice, 4T1 (C and E) and MC38 (D and F) TB mice were analyzed by flow cytometry for PD-L1 and other myeloid cells markers as indicated. Data are displayed as mean ± SEM with three independent experiments. *, $P<0.05$; **, $P<0.005$ by Welch's *t* test. (**G)** Representative images showing the CFSE gating of proliferating CD8^{+} T cells co-cultured with BM derived myeloid cells from NTB mice at different ratios.

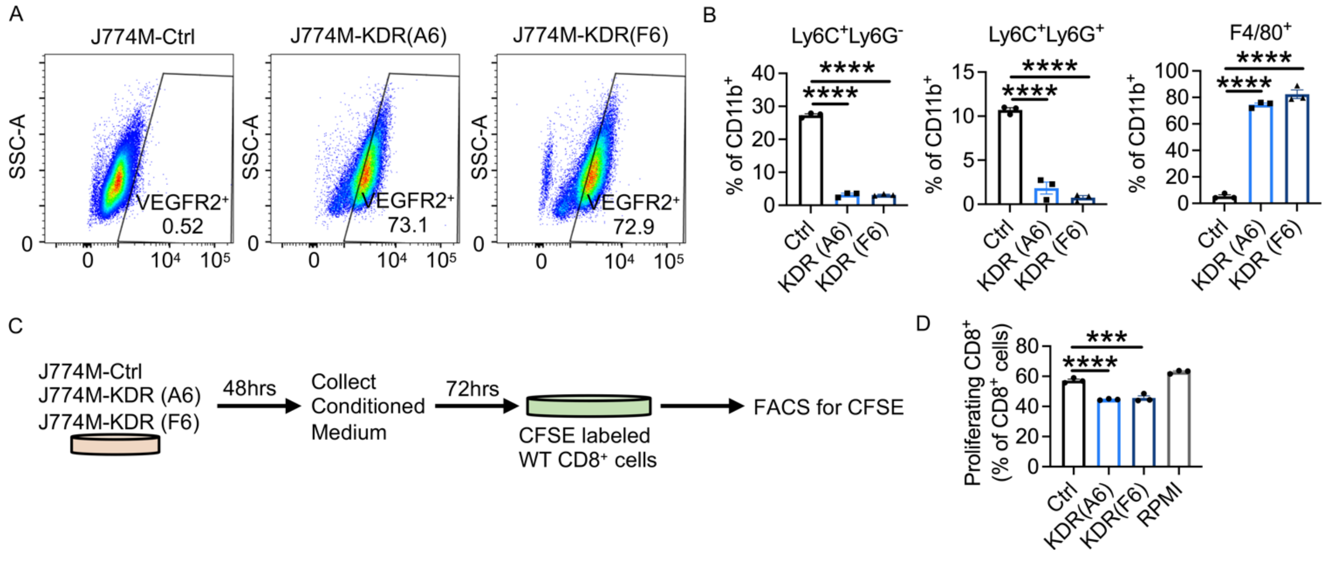

Supplementary Figure 3. Development of a VEGFR2 over-expression myeloid cell line. (A) Representative flow cytometry analysis of VEGFR2 expression in J774M-Ctrl, J774M-KDR (A6), J774M-KDR (F6) cells. (**B)** Flow cytometry analysis of the indicated markers expression on J774M-Ctrl, J774M-KDR (A6), J774M-KDR (F6) cells. Data are displayed as mean ± SEM with three independent experiments. ****, *P*<0.0001 by ANOVA with Tukey's MCT. (**C-D)** Conditioned media from 48hrs culture of J774M-Ctrl and J774M-KDR (A6), J774M-KDR (F6) cells were harvested and added to CFSE-labeled wild type CD8^{+} T cells (C). Percentage of proliferating CD8^{+} T cells after 72 hours was analyzed by flow cytometry (D). Data are displayed as mean ± SEM with three independent experiments. ***, *P*<0.001; ****, *P*<0.0001 by ANOVA with Tukey's MCT.

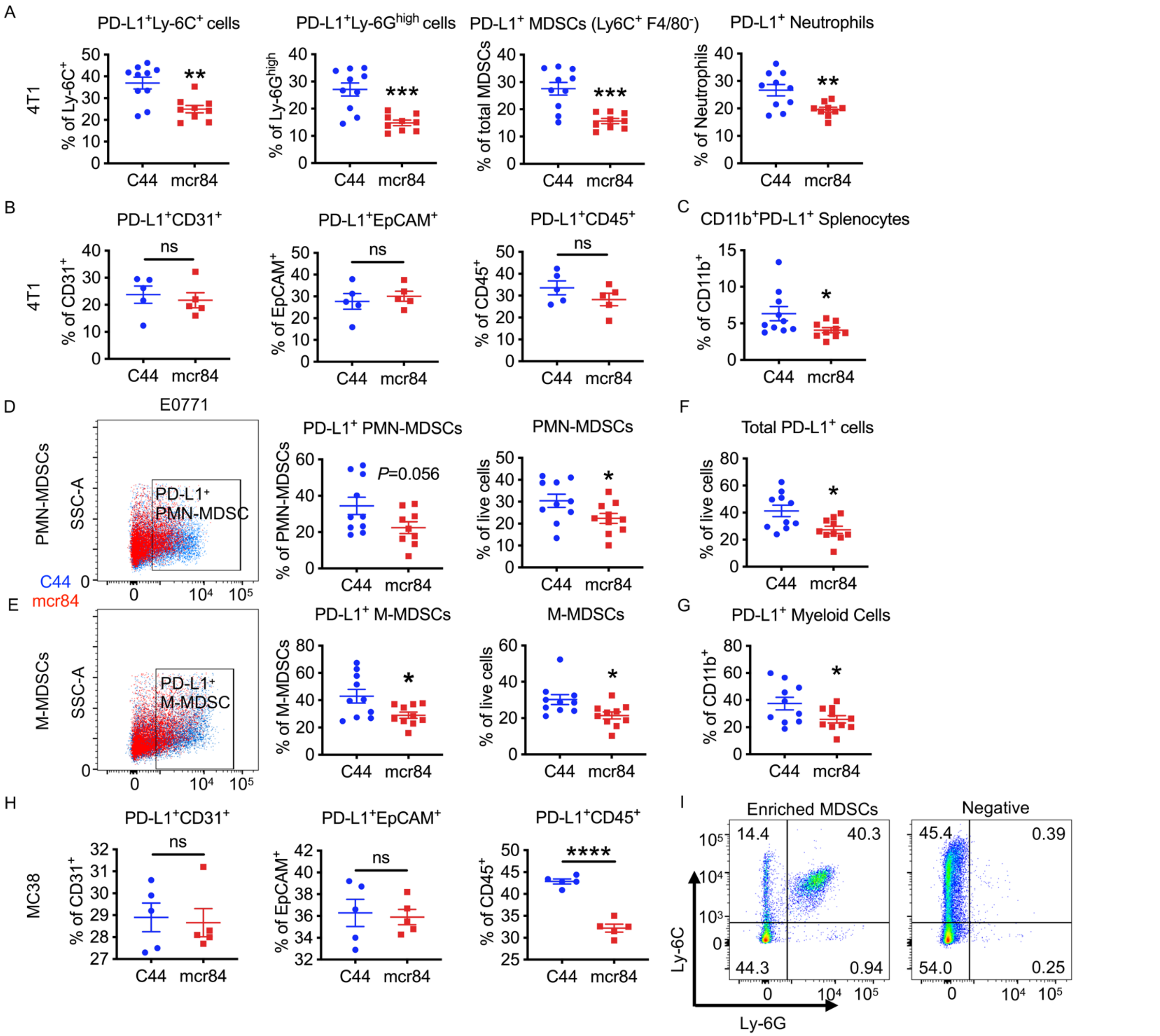

Supplementary Figure 4. VEGF blockade by mcr84 decreases PD-L1 expression specifically on myeloid cells. (A) Flow cytometry analysis of the indicated cell types in 4T1 tumors. Neutrophils were characterized as Ly-6G$^+$Ly-6C$^-$. Each dot indicates one tumor. PD-L1 expression was evaluated on each cell type. Data are displayed as mean ± SEM (n=9-10/group). **, *P*<0.005; ***, *P*<0.001, by Welch's *t* test. (**B**) PD-L1 expression was evaluated on indicated cell types in 4T1 tumors. Data are displayed as mean ± SEM (n=5/group). (**C**) Flow cytometry analysis of PD-L1 expression on total CD11b$^+$ myeloid cells in splenocytes of 4T1 tumor-bearing animals treated as indicated. Data are displayed as mean ± SEM (n=9-10/group). *, *P*<0.05 by Welch's *t* test. (**D-G**) Representative flow cytometry analysis of PD-L1 expression on gated PMN-MDSCs and M-MDSCs in E0771 tumors and flow cytometry analysis of the indicated cell types in E0771 tumors. Expression of PD-L1 on PMN-MDSCs (D), M-MDSCs (E) and total CD11b$^+$ myeloid cells (G) as well as total numbers of MDSCs (D-E), PD-L1$^+$ cells (F) were evaluated. Data are displayed as mean ± SEM (n=8-9/group). *, *P*<0.05 by Welch's *t* test. **(H)** PD-L1 expression was evaluated on indicated cell types in MC38 tumors. Data are displayed as mean ± SEM (n=5/group). ****, *P*<0.0001 by Welch's *t* test. **(I)** Representative flow cytometry analysis of enriched MDSCs sorted from tumors and negative components after sorting.

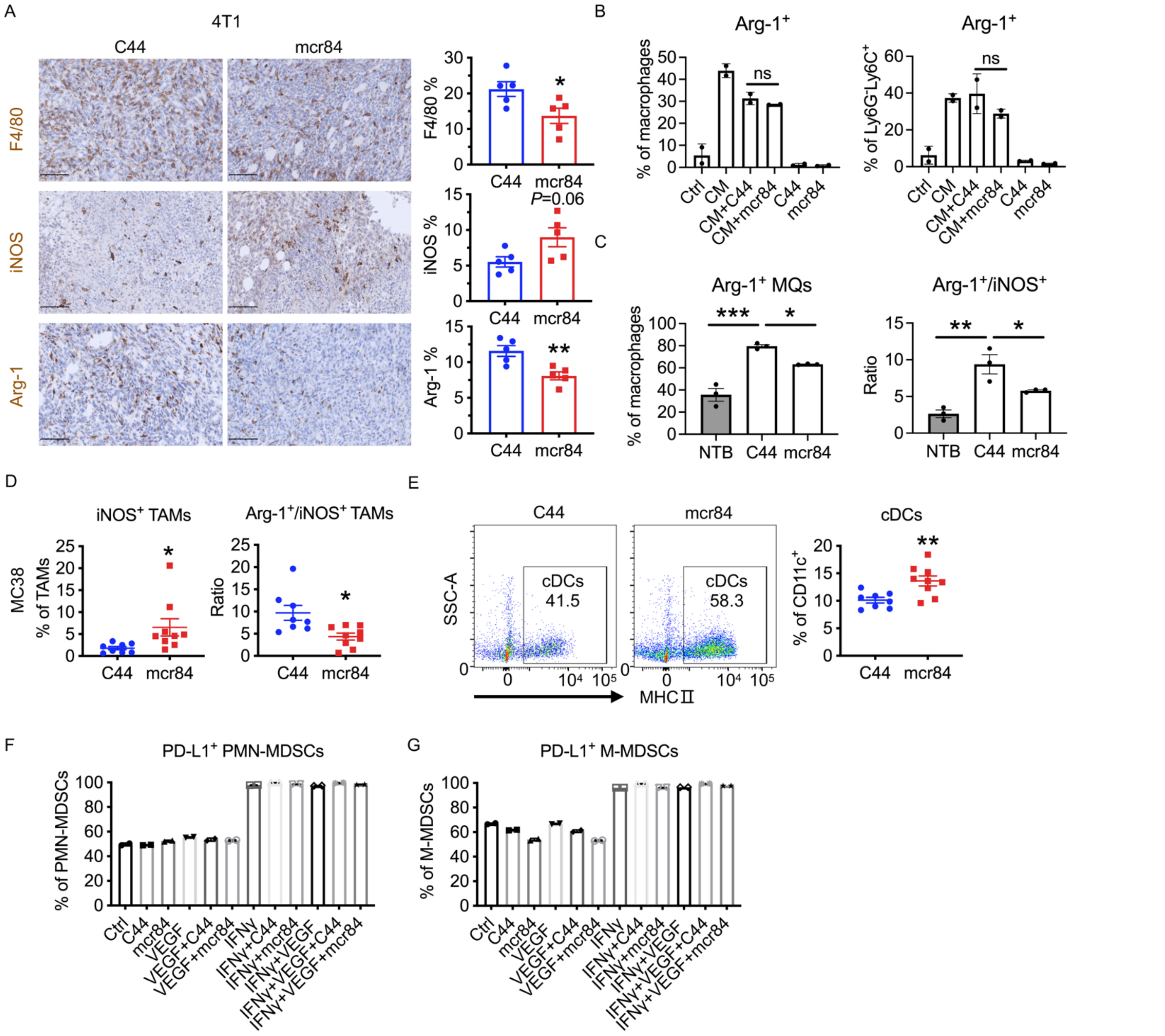

Supplementary Figure 5. VEGF blockade by mcr84 polarizes macrophages to an immunostimulatory phenotype. (A) Immunohistochemistry of FFPE 4T1 tumors for F4/80, iNOS, Arg-1. Slides were scanned and images were analyzed using Fiji software. Representative images and quantification are shown. Scale bar, 100 µm. Data are displayed as mean ± SEM (n=5/group). *, $P < 0.05$; **, $P < 0.05$ by Welch's *t* test. **(B)** 4T1 tumor cell conditioned medium (CM) or with C44/mcr84 were added to BM-MQs from NTB at Day 6. After 48 hours, BM-MQs were harvested and Arg-1 expression on indicated cell types was analyzed by flow cytometry. Data are displayed as mean ± SD with two independent experiments. (**C)** BM-MQs differentiated from NTB mice, 4T1 TB mice treated with C44 or mcr84 were analyzed for Arg-1 and iNOS expression by flow cytometry. Data are displayed as mean ± SEM with three independent experiments. *, $P<0.05$; **, $P<0.005$; ***, $P<0.001$ by ANOVA with Tukey's MCT. (**D-E)** Flow cytometry analysis of the indicated cell types in MC38 tumors. Tumor-associated macrophages **(TAMs)** (D) and cDCs (characterized as CD11c$^+$F4/80$^-$CD45R$^-$MHCII$^+$) (E) were evaluated. Data are displayed as mean ± SEM (n=8-9/group). *, $P < 0.05$; **, $P < 0.005$ by Welch's *t* test. (**F-G)** Bone marrow from C57BL/6 mice was harvested and differentiated into MDSCs in vitro. On Day 6, BM-myeloid cells were treated with VEGF +/- mcr84 or IFNγ+VEGF +/- mcr84 for 24 hours. MDSCs were analyzed by flow cytometry for PD-L1 expression. Flow cytometry analysis of PD-L1 expression on PMN-MDSCs and M-MDSCs are shown in (F-G). Data are displayed as mean ± SD with two independent experiments.

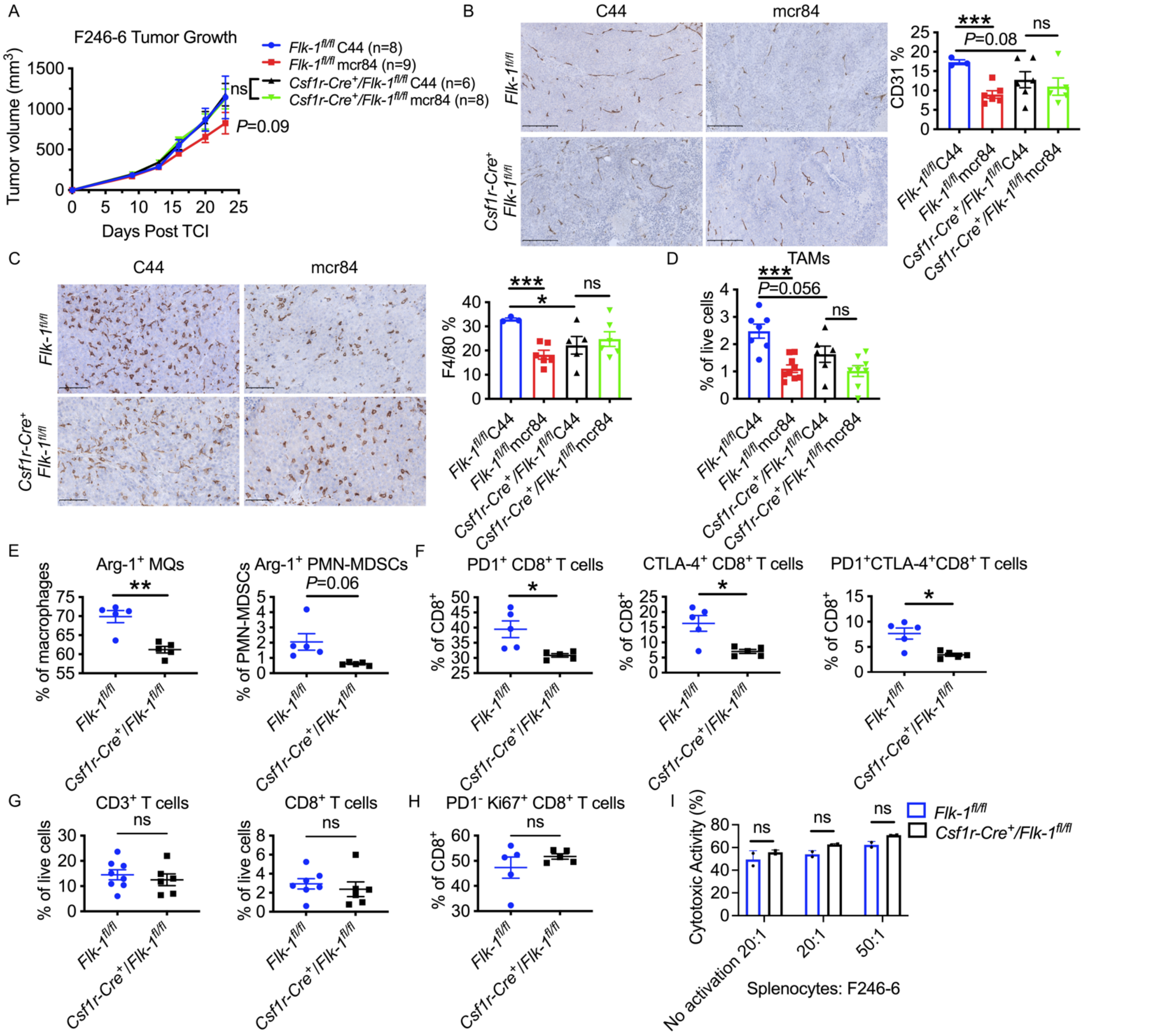

Supplementary Figure 6. Genetic deletion of *Flk-1* on myeloid cells recapitulates the effect of mcr84. (A) 5 × 10^5 F246-6 cells, a murine breast cancer cell line derived from MMTV-PyMT mice, were injected orthotopically into mammary fat pad of *Flk-1*$^{fl/fl}$ and *Csf1r-Cre*$^+$ *Flk-1*$^{fl/fl}$ mice. Mice with established tumors (50–150 mm^3) were treated with control antibody (C44, 250 μg/dose, twice per week) or mcr84 (250 μg/dose, twice per week) for 5 doses in total. Mice were monitored daily and tumor volume was measured twice per week. Tumor growth was analyzed (n=6-9/group). All mice were sacrificed at the same time and tumors were harvested. (**B-C)** Tumors were analyzed for CD31 and F4/80 by immunohistochemistry. Representative images are shown in the left panel. Scale bar, 100 μm. Data are displayed as mean ± SEM (n=3-6/group). *, $P < 0.05$; ***, $P < 0.001$, by Welch's *t* test. (**D)** Flow cytometry analysis of TAMs. Data are displayed as mean ± SEM with n=6-9 per group analyzed. ***, $P < 0.001$ by Welch's *t* test. (**E-H)** Flow cytometry analysis of indicated markers of myeloid cells and T cells in *Flk-1*$^{fl/fl}$ and *Csf1r-Cre*$^+$ *Flk-1*$^{fl/fl}$ F246-6 TB mice. Data are displayed as mean ± SEM with n=5-8 per group analyzed. *, $P < 0.05$; **, $P < 0.005$ by Welch's *t* test. (**I)** An in vitro cell cytotoxicity assay was performed following the instruction of the basic cytotoxicity assay kit. Splenocytes from *Flk-1*$^{fl/fl}$ and *Csf1r-Cre*$^+$ *Flk-1*$^{fl/fl}$ F246-6 TB animals were co-cultured with CFSE pre-labeled F246-6 tumor cells at different ratios for 72 hours and dead cells were labeled with 7-AAD. Samples were analyzed by flow cytometry. Data are displayed as mean ± SD with two independent experiments.

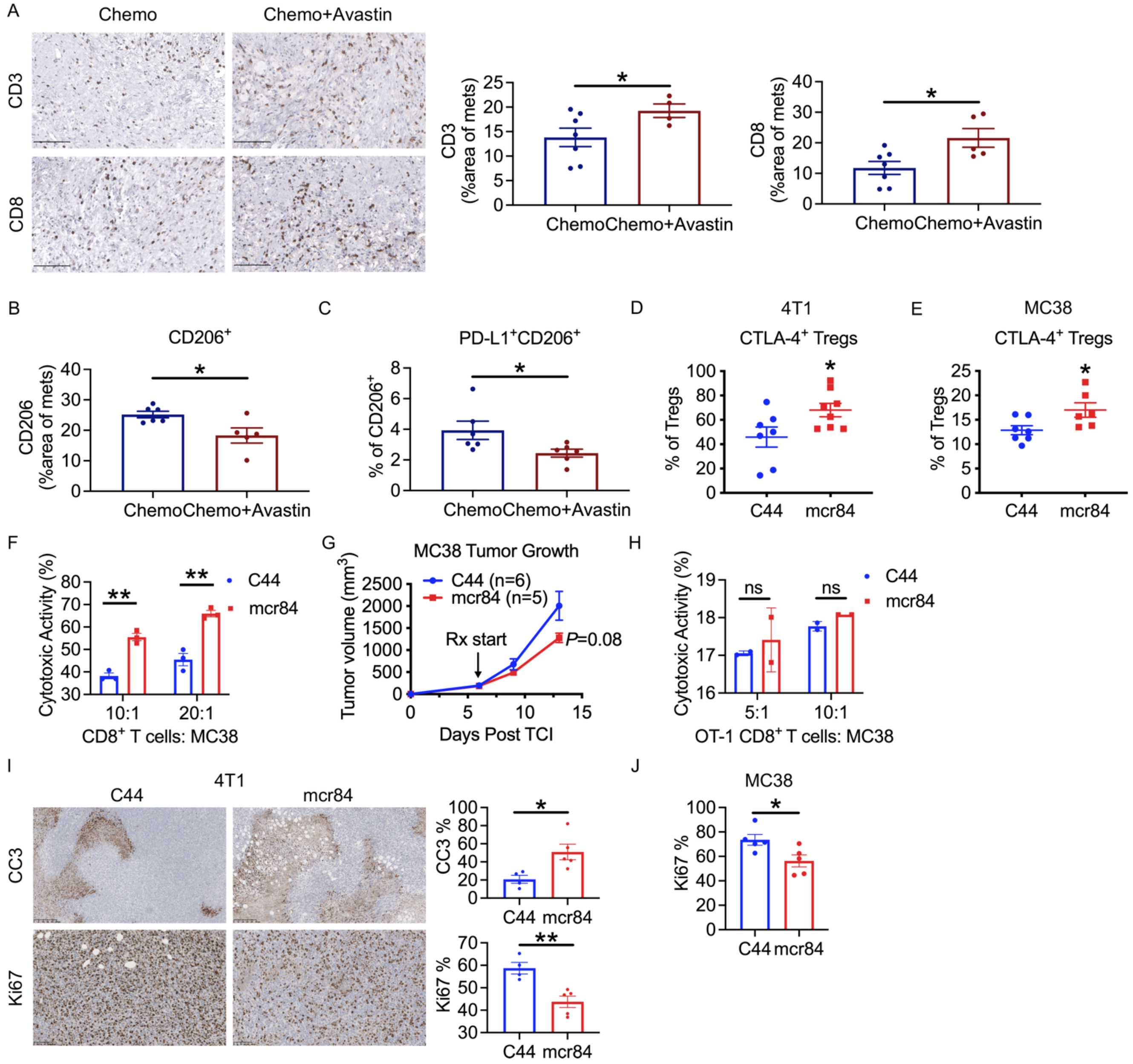

Supplementary Figure 7. VEGF blockade by mcr84 increases T cell infiltration and stimulates T cell activation. (A-C) Immunohistochemistry for CD3, CD8, CD206 and PD-L1 on liver tissues of colorectal cancer patients underwent chemotherapy or chemotherapy and avastin treatment. Slides were scanned and images were analyzed using NIS Elements (Nikon) and Fiji software. Representative images of CD3 and CD8 staining are shown. Scale bar, 250 μm. Each dot indicates one patient. Data are displayed as mean ± SEM (n=4-7/group). *, $P < 0.05$, by Welch's *t* test. (**D-E)** CTLA-4 expression on Tregs was evaluated by flow cytometry in 4T1 and MC38 tumors treated as indicated. Data are displayed as mean ± SEM. *, $P < 0.05$, by Welch's *t* test. (**F**) $CD8^+$ T cells from splenocytes of MC38 TB mice treated as indicated were co-cultured with CFSE pre-labeled MC38 cells for 72 hours and dead cells were labeled with 7-AAD. Samples were analyzed by flow cytometry. n =3/group. **, $P < 0.005$, by Welch's *t* test. (**G**) 1×10^5 MC38-OVA cells were injected subcutaneously into 6-week-old OT-1 mice (n=5-6/group). Mice with established tumors were treated with C44 or mcr84 (250 μg/dose, twice per week). Tumor volume was measured. Data are displayed as mean ± SEM. (**H)** $CD8^+$ T cells from splenocytes of OT-1 mice were co-cultured with CFSE pre-labeled MC38 cells for 48 hours and dead cells were labeled with 7-AAD. Samples were analyzed by flow cytometry. (**I-J)** FFPE 4T1 (I) and MC38 (J) tumors were assessed for cleaved caspase-3 (CC3) and Ki67. Slides were scanned and images were analyzed using Fiji software. Representative images of 4T1 model are shown. Scale bar, 250 μm or 100 μm. Data are displayed as mean ± SEM (n=5/group). *, $P < 0.05$; **, $P < 0.01$ by Welch's *t* test.
